# Repression of Flowering by the miR172 Target SMZ

**DOI:** 10.1371/journal.pbio.1000148

**Published:** 2009-07-07

**Authors:** Johannes Mathieu, Levi J. Yant, Felix Mürdter, Frank Küttner, Markus Schmid

**Affiliations:** Max Planck Institute for Developmental Biology, Department of Molecular Biology, Tübingen, Germany; John Innes Centre, United Kingdom

## Abstract

A small mobile protein, encoded by the *FLOWERING LOCUS T* (*FT*) locus, plays a central role in the control of flowering. *FT* is regulated positively by CONSTANS (CO), the output of the photoperiod pathway, and negatively by FLC, which integrates the effects of prolonged cold exposure. Here, we reveal the mechanisms of regulation by the microRNA miR172 target *SCHLAFMÜTZE* (*SMZ*), a potent repressor of flowering. Whole-genome mapping of SMZ binding sites demonstrates not only direct regulation of *FT*, but also of many other flowering time regulators acting both upstream and downstream of *FT*, indicating an important role of miR172 and its targets in fine tuning the flowering response. A role for the miR172/SMZ module as a rheostat in flowering time is further supported by SMZ binding to several other genes encoding miR172 targets. Finally, we show that the action of SMZ is completely dependent on another floral repressor, FLM, providing the first direct connection between two important classes of flowering time regulators, AP2- and MADS-domain proteins.

## Abstract 2

The flowering repressors SMZ and FLM, members of the AP-2 and MADS domain transcription factor families, unexpectedly work together to regulate flowering time via their effects on expression of the *FT* gene.

## Author Summary

Flowering is a pivotal event in the life cycle of many plants and is therefore under tight control. The ability to detect the daily photoperiod is of particular importance in many plant species, as it enables them to enter the reproductive phase in response to seasonal changes in day length. When the photoperiod is permissive to flowering, a signal is produced in leaves that is transported to the shoot meristem, where it initiates the formation of flowers. It is now widely accepted that an important component of this long-distance signal is the flowering protein FT. Here, we show that the AP2-like transcription factor SMZ, which represses flowering and is a target of the regulatory miRNA172 microRNA, functions together with related proteins to directly regulate *FT* expression. Using chromatin immunoprecipitation coupled to genome tiling arrays, we find that SMZ binds directly to the *FT* genomic locus and to several other key flowering-related loci. Unexpectedly, the ability of SMZ to repress flowering strictly depends on the presence of the MADS-domain transcription factor FLM. In addition, SMZ binds to its own regulatory sequences and those of three closely related genes, providing evidence of strong negative feedback between SMZ and the other AP2-like miRNA172 targets.

## Introduction

Throughout their lives, plants progress through distinct developmental phases, from germination and vegetative growth to flowering and, finally, senescence. The transition from vegetative growth to flowering is of particular importance because the correct timing of this switch is mandatory to ensure reproductive success. Plants have therefore evolved an elaborate genetic network that integrates endogenous and environmental signals to guarantee that flowering commences when conditions are most favorable.

Genetic and molecular analyses in *Arabidopsis thaliana* and other plants have identified several distinct genetic pathways that are involved in regulating the floral transition [1],[2]. On the basis of genetic interactions, one can distinguish between the gibberellic acid pathway, the autonomous pathway, and the vernalization pathway. Finally, light, and especially day length, is an important stimulus that is integrated into the flowering time regulatory network by the photoperiod pathway [3],[4]. *A. thaliana* is a facultative long-day plant, which means that it will flower more rapidly when day length exceeds a critical minimum. Interestingly, plants measure photoperiod in the leaves and not at the shoot apex where the new flowers will be formed. It has therefore been long postulated that the light-exposed leaves produce a flower-forming substance to regulate the formation of flowers at the shoot apex [5],[6]. This ultimately led to the formation of the “florigen” hypothesis, which postulated that a substance, “florigen,” is produced in leaves under inductive photoperiod and is transported to the shoot apex to induce flowering [7]. It was later demonstrated that such a flower-inductive substance could be transmitted from one plant (donor) via grafting to another plant (receptor) that had been cultivated under noninductive conditions.

An important factor that allows *Arabidopsis* to discriminate between short day (SD) and inductive long day (LD) is the B-box–type zinc finger protein CONSTANS (CO) [8]. The regulation of CO at both the mRNA and protein levels ensures that the protein will accumulate and activate flowering only under LD conditions [4],[9],[10]. Interestingly, CO appears to carry out its function in leaves, where it acts in the phloem companion cells to regulate a systemic signal that induces photoperiodic flowering [11],[12].

Several lines of evidence suggest that the protein FLOWERING LOCUS T (FT) acts as a florigen to convey flowering time signals from the leaves to the apex [13],[14]. First, it was established that *FT* is the major target of CO in leaves [15],[16]. It was further demonstrated that the FT protein interacts at the shoot apex with another flowering time regulator, the bZIP transcription factor FD, to induce downstream flower-specific targets such as the MADS-domain proteins *APETALA1* (*AP1*) and *FRUITFULL* (*FUL*) [16],[17]. The finding that *FT* is transcribed in leaves but acts at the apex implied that FT can move, either as mRNA or as protein. Later experiments were unable to detect *FT* mRNA movement but provided evidence that FT protein is able to reach the apex when expressed in the vasculature [18]–[23].

Interestingly, the induction of flowering under LD by CO/FT is counteracted by several factors that either prevent *FT* expression in the leaf or act downstream of FT to modulate its function at the shoot apex. In particular, MADS-domain transcription factors have been shown to act as repressors of flowering. The most prominent of these is FLOWERING LOCUS C (FLC), which represses flowering in winter annual accessions of *Arabidopsis* before the plants have been exposed to a prolonged period of cold [24]. It has recently been shown that FLC, when expressed either from the phloem-specific *SUC2* promoter or the meristem-specific *KNAT1* promoter, efficiently represses flowering and that these effects are additive. Further, it was demonstrated that FLC directly binds to the regulatory regions of three positive regulators of flowering, *FT*, *FD*, and *SUPRESSOR OF OVEREXPRESSION OF CONSTANS 1* (*SOC1*), presumably to repress these genes [25]. Two other MADS-domain transcription factors, FLM and SVP, have also been shown to repress flowering. In contrast to FLC, which is involved in the vernalization pathway, these two genes seem to be involved predominately in the photoperiod pathway, and *FLM* and *SVP* act as partners [26]–[28]. There is, however, also evidence that implicates SVP and FLM in temperature-dependent regulation of flowering in *Arabidopsis*, and SVP has recently been shown to interact with FLC in a repressor complex [29]. In addition, SVP has also been shown to directly bind to regulatory regions of *FT* and *SOC1* [29],[30].

More recently, two more transcription factors, *TEMPRANILLO 1* (*TEM1*) and *TEM2*, have been shown to redundantly repress flowering [31]. In contrast to FLC, FLM and SVP, which are MADS-domain transcription factors, each *TEM* gene encodes an AP2 domain as well as a B3-type DNA binding domain. *TEM1* is most strongly expressed in leaves, where its expression is regulated in a circadian fashion [31]. TEM1 was further shown to directly bind to the 5′ UTR of *FT* [31]. This is in contrast to FLC, which bound most strongly to the first intron of *FT*, indicating that *FT* is regulated by different repressors in different regions.

Yet another family of six AP2-like transcription factors also act as repressors of flowering. This clade of proteins comprises APETALA 2 (AP2) itself, the three TARGET OF EAT (TOE) proteins (TOE1, TOE2, and TOE3), and SCHLAFMÜTZE (SMZ) and its paralog SCHNARCHZAPFEN (SNZ) [32]–[34]. All six genes have in common that they are predicted targets of microRNA172 (miR172), expression of which is regulated by GIGANTEA (GI) to control flowering in a CO-independent manner [35]. It has previously been shown that TOE1 and TOE2 act as repressors of flowering: *toe1* mutants are significantly early flowering, and this effect is enhanced in a *toe1 toe2* double mutant [33],[35]. However, plants that expressed miR172 constitutively were found to flower much earlier than even the *toe1 toe2* double mutant, indicating that the other AP2 family members most likely act redundantly with TOE1 and TOE2 to repress flowering [33],[36]. A good candidate for such a repressor is SMZ, which was originally identified in an activation-tagging screen because of its dominant late-flowering phenotype [34]. Additionally, SNZ, a paralog of SMZ, has been shown to repress flowering when expressed at high levels [34]. However, it was unclear whether SMZ and SNZ normally act as repressors of flowering.

Here, we show that the miR172 targets *SMZ* and *SNZ* are bona fide floral repressors and act redundantly with *TOE1* and *TOE2* to delay flowering specifically under LD conditions. Plants expressing *SMZ* at high levels are late flowering, which is due to an almost complete block in *FT* induction. The effects of SMZ on *FT* expression appear to be direct, as chromatin immunoprecipitation coupled to hybridization to tiling arrays (ChIP-chip) identified *FT* as a target of SMZ. In addition, several other known regulators of flowering time were identified as SMZ targets. Among them are *SMZ* itself, *SNZ*, *AP2*, and *TOE3*, suggesting a complex feedback regulation among miR172 targets. Finally, we found that repression of flowering by SMZ is independent of the potent floral repressor FLC, but requires FLM for its function, providing a direct connection between two important classes of flowering time regulators, AP2- and MADS-domain proteins.

## Results

### SMZ Represses Floral Induction by Inhibiting the Photoperiod Pathway

**Figure 1:**
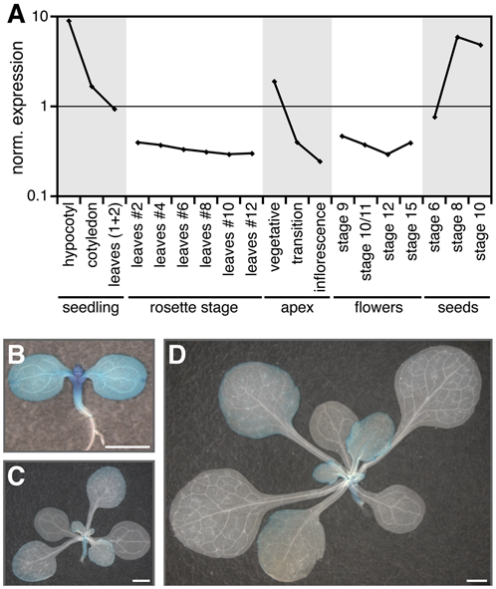
Expression pattern of *SMZ*. (A) Expression of *SMZ* is developmentally regulated. High *SMZ* expression is detected in hypocotyl, cotyledons, and the meristematic region of 7-d-old seedlings (data from AtGenExpress expression atlas [41], selected samples). *SMZ* is not detectable in leaves at the rosette stage and flowers, but *SMZ* mRNA levels increase again as seeds mature. Nomenclature of floral and seed stages according to [63] and [64], respectively. (B–D) A gSMZ∶GUS reporter confirms the developmental regulation of SMZ in 5-d-old (B), 10-d-old (C), and 15-d-old (D) seedlings. Scale bar indicates 1 mm.

*smz-D* was originally isolated as a dominant late-flowering mutant in an activation-tagging screen under LD conditions. *SMZ* is expressed in young seedlings and is developmentally regulated, as deduced from microarray data (Figure 1A) and confirmed by a genomic SMZ∶GUS reporter (Figure 1B–1D). Expression of SMZ declines with increasing age, but SMZ is induced again in seeds during maturation. In addition, analysis of publicly available microarray data (“The diurnal project”; http://diurnal.cgrb.oregonstate.edu/) revealed that *SMZ* exhibits a diurnal expression with a maximum at Zeitgeber 15 under LD conditions [37].

**Figure 2:**
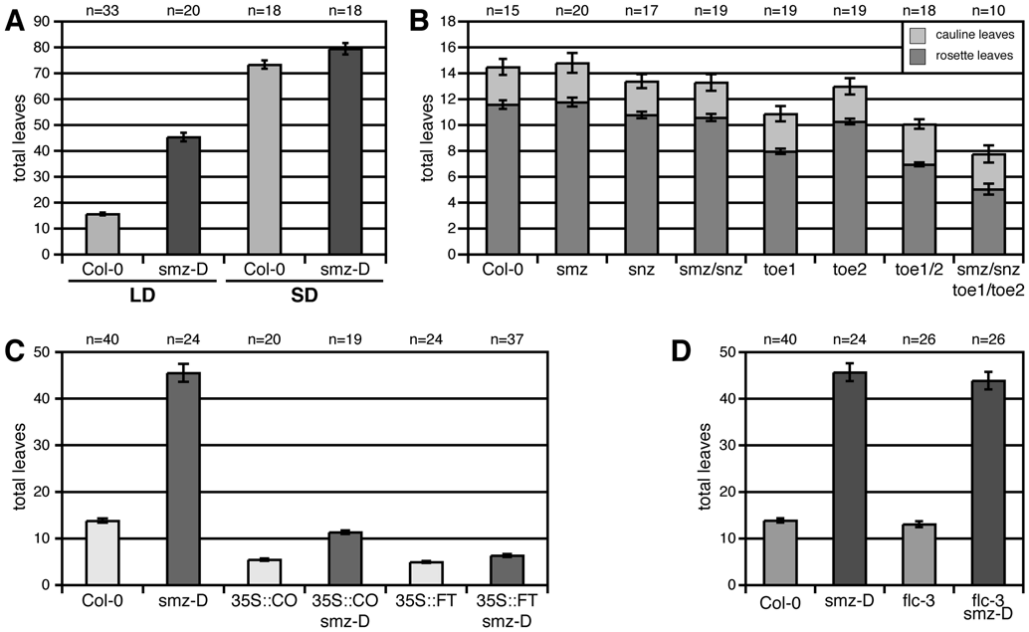
Genetic interactions of SMZ. (A) SMZ acts as a repressor of flowering under LD photoperiod. *smz-D* plants flower late under LD, but normally under SD conditions. (B) SMZ and SNZ act redundantly with TOE1 and TOE in controlling floral transition. Loss-of-function alleles of *smz-2* and *snz-1* significantly (*p*<0.001) enhance the early flowering of a *toe1-2 toe2-1* double mutant. Number of rosette leaves (dark grey) and cauline leaves (light grey) are shown. (C) *smz-D* delays flowering in *35S::CO*. *smz-D* partially represses early flowering caused by *CO* overexpression. Early flowering by overexpression of *FT* is not alleviated by *smz-D*. (D) SMZ represses flowering independently of FLC. *smz-D* was introduced into the *flc-3* background. Double homozygous plants and controls were grown under LD conditions. Error bars indicate 2× standard error of the mean (SEM).

To better understand where *SMZ* functions in respect to the known flowering time pathways, we first investigated the flowering time behavior in this mutant under different day lengths (Figure 2A and Table 1). We found that *smz-D* delays the onset of flowering specifically under inductive LD conditions, where it produced 45.1±1.7 leaves before flowering compared to wild-type (15.5±0.6 leaves). In contrast, under noninductive SD conditions, *smz-D* (79.1±2.2 leaves) plants flowered similarly to wild-type (73.0±1.6 leaves), indicating that *smz-D* represses flowering specifically under inductive LD, but has little effect under SD conditions.

**Table 1:** Flowering time of *smz-D* and *smz* loss-of-function mutants.

Experiment	Genotype	Leaves	Deviation	Range	*n*
**1 (23°C, LD)**	Col-0	15.5	0.6	13–19	33
	*smz-D*	45.1	1.7	38–51	20
**2 (23°C, SD)**	Col-0	73.0	1.6	67–77	18
	*smz-D*	79.1	2.2	63–78	18
**3 (23°C, LD)**	Col-0	14.3	0.6	13–17	15
	*smz-2*	14.6	0.8	10–18	20
	*snz-1*	13.3	0.5	11–15	17
	*smz-2 snz-1*	13.2	0.6	10–15	19
	*toe1-2*	10.8	0.6	8–14	19
	*toe2-1*	12.8	0.6	9–15	19
	*toe1-2 toe2-1*	10.0	0.4	9–11	18
	*smz-2 snz-1 toe1-2 toe2-1*	7.7	0.7	6–10	10
**4 (23°C, LD)**	Col-0	13.8	0.5	11–16	40
	*smz-D*	45.6	1.9	38–56	24
	*35S::CO*	5.4	0.3	5–7	20
	*smz-D 35S::CO*	11.3	0.4	10–12	19
	*35S::FT*	4.9	0.2	4–6	24
	*smz-D 35S::FT*	6.3	0.3	5–8	37
	*flc-3*	13.0	0.6	11–16	20
	*smz-D flc-3*	43.8	1.9	35–50	26

For each genotype, the mean of the total leaf number, the deviation from the mean (2× the standard error of the mean), the range of values found for each genotype, and the number of plants examined are given.

LD, long day; SD, short day.

### SMZ and SNZ Are Repressors of Flowering

To investigate whether *SMZ* is indeed functioning as a floral repressor, we isolated homozygous *SMZ* loss-of-function alleles from T-DNA insertion collections. Neither of the individual *smz* mutant lines displayed any obvious phenotypes; in particular, the total number of leaves did not significantly differ from that of wild-type plants (Figure 2B and Table 1). Most notably, the plants were not early flowering, as one would have expected to result from the loss of a putative floral repressor. Also, a double mutant lacking *SMZ* and its closest paralog *SNZ* was found to be indistinguishable from the wild type (Figure 2B).

Together with *AP2*, *TOE1*, *TOE2*, and *TOE3*, *SMZ* and *SNZ* form a clade of six AP2-domain transcription genes. Because functional redundancy has been observed within this clade in respect to the timing of floral induction of the *toe1 toe2* double mutant, we first focused on the function of *TOE1*, *TOE2*, *SMZ* and S*NZ* rather than that of *AP2* and *TOE3*, which are predominately expressed at the meristem, and created a mutant line that lacks *toe1 toe2 smz snz* functions. This quadruple mutant was found to flower significantly earlier than Col-0, *toe1*, and even *toe1 toe2* double-mutant plants (Figure 2B and Table 1; *p*<0.001 in all comparisons). This result confirms that SMZ and its paralog SNZ are indeed acting as floral repressors redundantly with TOE1 and TOE2. This effect is only apparent in the sensitized *toe1 toe2* mutant background, as *TOE1* normally masks the effects of *smz* and *snz* loss of function.

The early flowering we observed in certain combinations of *smz*, *snz*, *toe1*, and *toe2* loss-of-function alleles was associated with a reduced number of rosette leaves, whereas the number of cauline leaves remained constant (Figure S1). It is interesting to note that even the *toe1 toe2 smz snz* quadruple mutant still flowers significantly later than plants that constitutively express miR172, which have been reported to produce on average two to three rosette leaves before bolting [33],[36]. This strongly suggests that the two remaining miR172 targets, *AP2* and *TOE3*, also act to repress flowering, which is especially interesting given that these two genes are predominately expressed at the meristem.

We conclude from these results that SMZ and its homolog SNZ are bona fide floral repressors that, partly redundant with other members of the miR172 target family, act to delay flowering in *A. thaliana* under LD conditions.

### *smz-D* Attenuates Early Flowering in *35S::CO*

Two genes play key roles in the photoperiod pathway: *CO*, which constitutes the main readout of the circadian clock, and *FT*, which has been shown to be an important part of the mobile signal that conveys the information to induce flowering from the leaves to the apex [18]–[23].

To test the genetic position of *SMZ* in relation to these two factors, we introduced *smz-D* into established plant lines that expressed *CO* or *FT* under the control of the constitutive 35S promoter (Figure 2C). Lines expressing either of these two genes at a high level are extremely early flowering. We observed a substantial delay in flowering in *smz-D 35S::CO* plants (11.3±0.4 leaves) compared to the *CO* overexpressing line (5.4±0.3 leaves) (Figure 2C and Table 1). In contrast, *smz-D* had a much smaller effect on the flowering of plants expressing *FT* at high levels (6.3±0.3 leaves; compared to 4.9±0.2 leaves observed in *35S::FT*). These findings are compatible with the idea that SMZ acts as a repressor of flowering and counteracts the flower-promoting activity of CO.

### SMZ Represses Flowering Independently of FLC

Next, we tested the dependence of SMZ on the presence of functional FLC, as FLC is a well-described repressor of flowering, integrating environmental signals such as vernalization and ambient temperature [24].

FLC has been shown to directly bind to regulatory sequences of the *FT* gene as well as to the promoters of *SUPRESSOR OF OVEREXPRESSION OF CONSTANS 1* (*SOC1* = *AGL20*) and *FD* [29]. We therefore tested whether *smz-D* acts through *FLC* to repress flowering. When we introduced *smz-D* into the strong *flc-3* deletion mutant background, which lacks part of the 5′UTR and the first exon, and is a genetic null allele of *FLC*, we observed no difference in flowering time in *smzD flc-3* (43.8±1.9 leaves) when compared to *smz-D* (45.6±1.9 leaves), showing that *smz-D* represses flowering independently of *FLC* (Figure 2D and Table 1).

### *SMZ* and *SNZ* Are Targets of miR172

As mentioned above, *SMZ* and *SNZ* share a miR172 target site. To test whether the mRNAs of these two genes are indeed targeted for degradation and are cleaved at the predicted positions, we carried out RACE-PCR to map the 5′ end of miR172 cleavage products. We found that the *SMZ* mRNA was cleaved at the predicted site in all clones analyzed (*n* = 12; Figure S2). Similarly, correct cleavage of *SNZ* mRNA was observed in 12/13 (Figure S2) cases, confirming that both *SMZ* and *SNZ* are indeed miR172 targets.

**Figure 3:**
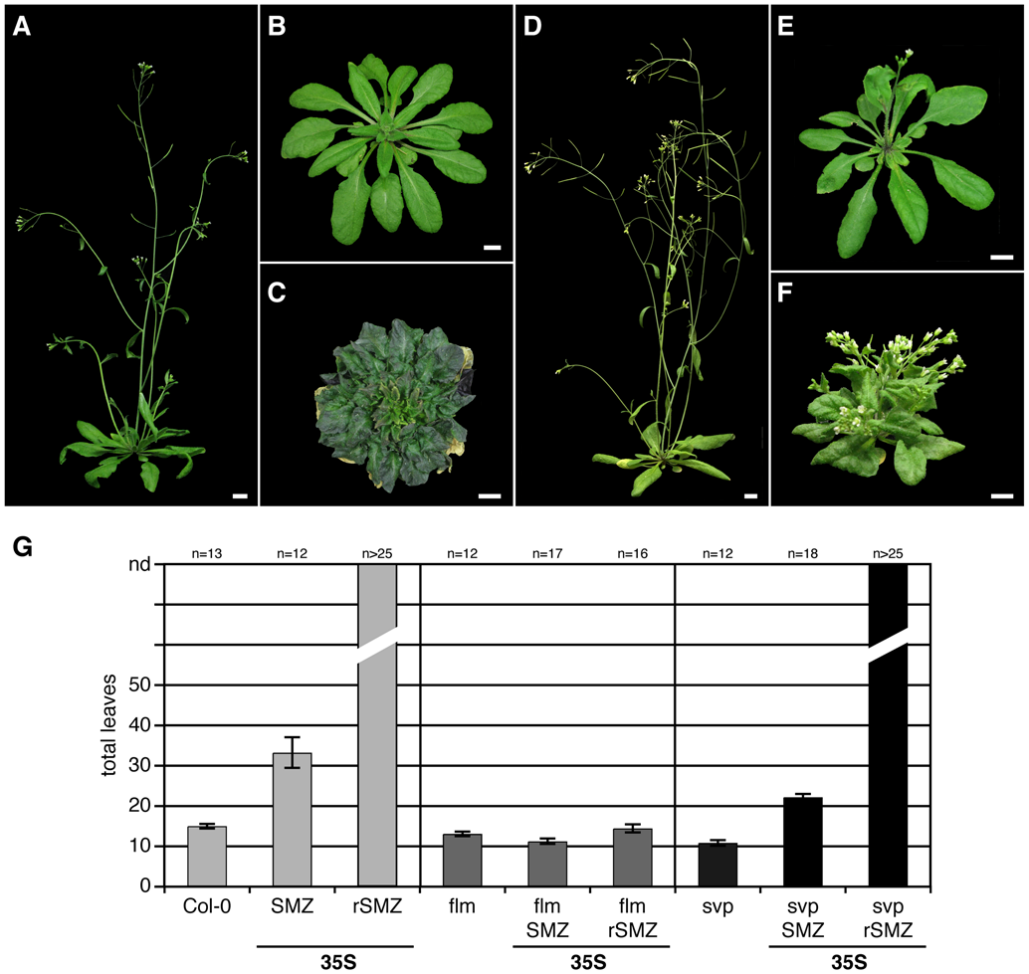
Repression of flowering by SMZ requires FLM. Early flowering of *flm-3* is epistatic over *SMZ* and *rSMZ* overexpression. Constitutive expression of *SMZ* (B and G) and *rSMZ* (C and G) in Col-0 background strongly delays the onset of flowering (G) compared to wild-type control (A and G). Expression of *SMZ* (E and G) and *rSMZ* (F and G) in a *flm-3* mutant background (D and G) results in wild-type–like flowering. (F) *rSMZ flm-3* plants display stunted growth, leaf curling, and reduced apical dominance. (G) Loss of *SVP* does not prevent late flowering by expression of 35S::(r)SMZ. Error bars indicate 2× SEM. Scale bars indicate 1 cm.

To further investigate whether the regulation of *SMZ* by miRNA172 plays a role in controlling the floral transition, we introduced into plants a version of *SMZ* mRNA (*rSMZ*) that carried silent mutations in the miR172 complementary site, rendering the mRNA resistant to miR172-directed cleavage (Figure S2). Strong expression of *rSMZ* from the constitutive 35S promoter caused plants to remain vegetative throughout their life (Figure 3C and 3G, and Table 2). In addition, the leaves of these plants displayed a crinkled phenotype and remained smaller than those of either wild-type controls or plants transformed with the *SMZ* ORF. The failure of *35S::rSMZ* plants to initiate flowering suggests that miR172-directed cleavage of *SMZ* mRNA in *smz-D* and *35S::SMZ* plants limits the effects of overexpressing the native version of *SMZ*.

**Table 2:** Effects of misexpression of *SMZ* on flowering.

Experiment	Genotype	Leaves	Deviation	Range	*n*
**1 (23°C, LD)**	Col-0	14.9	0.6	13–17	13
	*35S::SMZ* (line #1)	33.1	3.8	26–40	12
	*35S::rSMZ* a	>80	n/d	>80	>25
	*flm-3*	12.9	0.6	11–14	12
	*flm-3 35S::SMZ* (line #8)	11.1	0.7	8–14	17
	*flm-3 35S::rSMZ* (line #11)	14.3	1.0	11–18	16
	*svp-31*	10.7	0.7	8–12	12
	*svp-31 35S::SMZ* (line #16)	21.9	1.0	17–24	18
	*svp-31 35S::rSMZ* a	>80	n/d	>80	>25
**2 (23°C, LD)**	Col-0	13.8	0.6	12–17	12
	*35S::SMZ*	43.4	4.7	32–51	20
	*35S::rSMZ* a	>80	n/d	>80	>25
	Col-0	14.6	0.6	12–17	20
	*SUC2::SMZ*	46.7	6.5	20–61	19
	*SUC2::rSMZ*	44.7	7.4	13–68	18
	*FD::SMZ*	16.4	1.2	12–21	20
	*FD::rSMZ* a ^,^ b	28.0	7.33	11–69	17

For each genotype, the mean of the total leaf number, the deviation from the mean (2× the standard error of mean), the range of values found for each genotype, and the number of plants examined are given.

a Data were collected using transgenic plants in the T2 generation, except for *35S::rSMZ* in either wild-type or *svp-31* background where T1 data are shown because these genotypes did not induce flowering and/or did not produce any seeds.

b The increased number of cauline leaves in *FD::rSMZ* (up to 53 cauline leaves) contorts flowering time measurements in this genotype. The number of rosette leaves is only mildly increased in *FD::rSMZ* (14.2±1.5) compared to Col-0 (11.8±0.6).

LD, long day; n/d, not determined; SD, short day.

### SMZ Requires FLM to Repress Flowering

Besides FLC, two other MADS-domain proteins, SHORT VEGETATIVE PHASE (SVP) and FLOWERING LOCUS M (FLM = MAF1), have been shown to function as floral repressors [26],[27],[30]. On the basis of genetic analysis of mutant alleles, it has been suggested that FLM and SVP act as coregulated partners in the same pathway [28].

To test whether either of these two genes is required for SMZ function, *35S::SMZ* and *35S::rSMZ* constructs were transformed into established *svp* and *flm* T-DNA mutant lines. Loss of *SVP* did not affect the *SMZ* overexpression phenotype, i.e., *svp* plants carrying either the *35S::SMZ* or the *35S::rSMZ* transgene flowered just as late as control plants (Figure 3G and Table 2).

In contrast, the late flowering, which usually would result from *SMZ* overexpression, was completely abolished in the *flm* mutant background (Figure 3E and 3G, and Table 2). Even constitutive expression of the miR172-resistant form of *SMZ* (*rSMZ*) was no longer able to delay flowering (Figure 3F and 3G, and Table 2) in *flm* mutants. This is in contrast to the extreme effect of (r)SMZ on flowering in wild-type control transformations (Figure 3B, 3C, and 3G, and Table 2). Interestingly, we also observed that expression of *rSMZ* in *flm* resulted in reduced growth and crinkly leaves similar to what we had observed in wild-type control plants transformed with *35S::rSMZ* (Figure 3C), despite the fact that the plants now flowered with a normal number of leaves. In addition, apical dominance was reduced in these lines, giving the plants a bushy appearance. The fact that expression of *rSMZ* causes phenotypes even though no flowering time defects were observed rules out that the transgene was silenced in the *flm* background. In addition, levels of transgene expression were found to be comparable in Col-0 and *flm* background when analyzed by quantitative reverse transcription PCR (qRT-PCR) (unpublished data).

Because high levels of *FLM* have been shown to delay flowering, we therefore examined the expression of *FLM* in *smz-D* by qRT-PCR, but did not find any evidence that *FLM* levels were increased (unpublished data). Therefore, it does not appear that SMZ is simply up-regulating *FLM* transcription.

### SMZ Acts Primarily in Leaves to Regulate Flowering Time

Our previous results demonstrated that SMZ acts as a floral repressor in the photoperiod pathway. A characteristic of this pathway is the spatial separation of the perception of inductive photoperiod in the leaves and the formation of flowers at the shoot apical meristem. Recently, the FT protein has been shown to be an important component of the signal that transmits flowering time information from the leaves to the apex [18]–[23]. In the course of these studies, it was shown that for *FT* to exert its function in the photoperiod pathway, it is both necessary and sufficient for this gene to be expressed in leaf phloem companion cells [19],[20],[22]. Therefore, if the function of SMZ in flowering time is indeed to negatively regulate *FT* expression, misexpression of SMZ in phloem companion cells and the resulting repression of *FT* in this tissue should be sufficient to recapitulate the late-flowering phenotype observed in *smz-D*.

**Figure 4:**
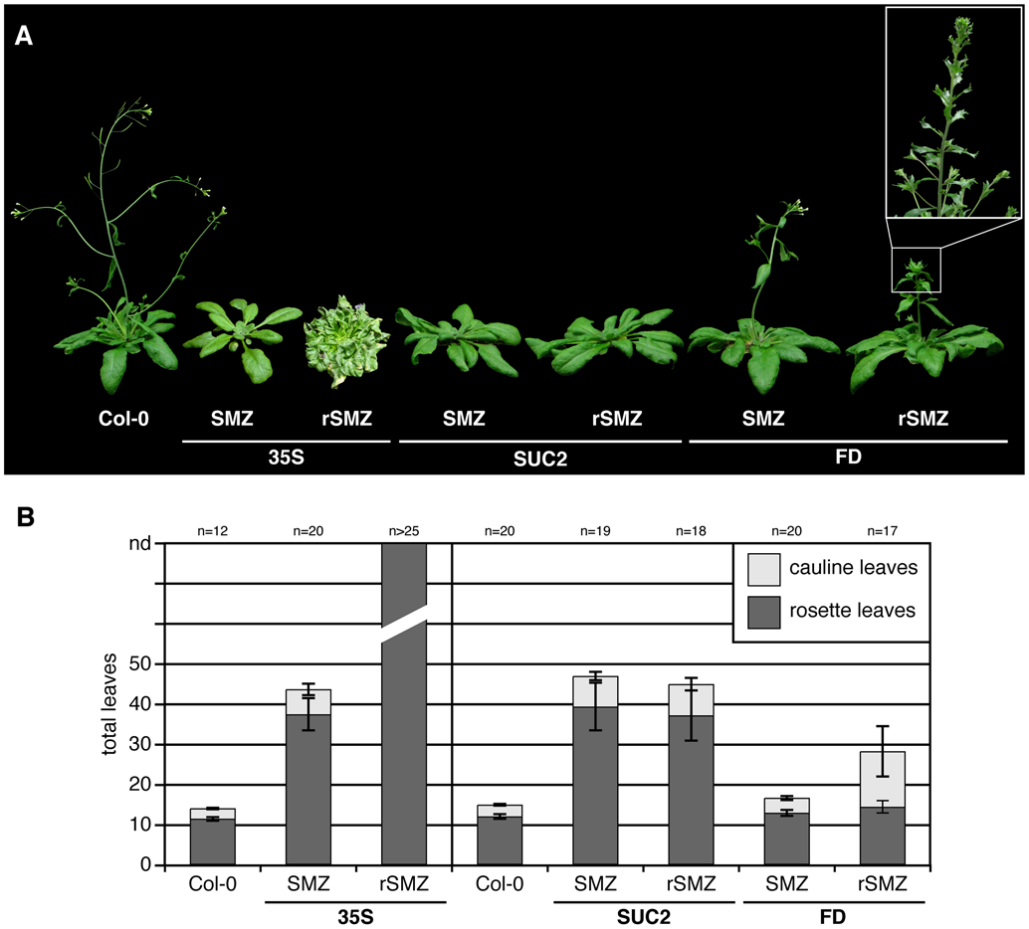
Tissue-specific misexpression of *SMZ* and *rSMZ*. (A) Phenotypes of plants expressing either *SMZ* or *rSMZ* mRNA under the control of the constitutive *35S* promoter, the phloem companion cell-specific *SUC2* promoter, or the meristem-specific *FD* promoter. Magnification of the abnormal shoot and flower morphology observed in *FD::rSMZ* plants is shown (inset; picture taken at a later time point). (B) Flowering time of *SMZ* and *rSMZ* misexpression plants. Data are from T2 plants, except for those lines that did not produce any flowers in T1 (*35S:: rSMZ*) or that did not produce any fertile flowers (*FD::rSMZ*) and for which T1 data are shown instead. Error bars indicate 2× SEM.

As expected, expression of *SMZ* from the phloem companion cell-specific *SUC2* promoter efficiently delayed flowering and resulted in late-flowering plants that were phenotypically indistinguishable from *35S::SMZ* plants (Figure 4A and 4B, and Table 2). Similarly, *rSMZ* driven from the phloem-specific promoter SUC2 did not cause plants to flower later than the miR172-susceptible *SMZ* ORF driven from the same promoter (Figure 4A and 4B, and Table 2). Furthermore, *SUC2::rSMZ* plants did not display any of the additional defects in leaf or shoot morphology that were evident in *35S::rSMZ* (Figure 4A), indicating that these phenotypes were caused by misexpression of *rSMZ* in tissues other than the vasculature. An alternative explanation could be that miR172 is normally not expressed in phloem companion cells, in which case, SMZ and rSMZ overexpression would have similar effects. This seems unlikely, however, as miR172 has been cloned from phloem exudates in *Brassica* [38].

In contrast, expression of *SMZ* from the shoot meristem-specific *FD* promoter had only the most modest effect on bolting time (Figure 4A and 4B, and Table 2). Even in *FD::rSMZ* plants, the number of rosette leaves (14.2±1.5) was similar to that in controls, indicating that high levels of SMZ at the shoot apex are not sufficient to delay the onset of flowering. The number of cauline leaves, however, was vastly increased in *FD::rSMZ* plants (13.8±6.3) compared to wild type (2.9±0.2) (Figure 4B and Table 2). Additionally, these plants displayed a shoot phenotype reminiscent of a double mutant lacking both the meristem identity gene *LEAFY* (*LFY*) and *AP1*, in that flowers were replaced by leaf-like organs, which were frequently subtended by bracts (Figure 4A). Taken together, these results suggest that SMZ can affect different sets of target genes in leaves and at the shoot meristem. An alternative explanation would be that the *FD* promoter becomes active too late in development to delay flowering but in time to repress flower development, causing this shoot phenotype.

### SMZ Represses *FT* Transcription

As we showed earlier, genetic analyses clearly place SMZ in the photoperiod pathway and tissue-specific misexpression of SMZ suggests that regulation of flowering time by SMZ occurs predominantly in the leaves. To analyze the molecular cause for the late flowering of SMZ overexpressing plants, we carried out quantitative RT-PCR on the putative target gene *FT*, which is normally induced in leaves under LD.

**Figure 5:**
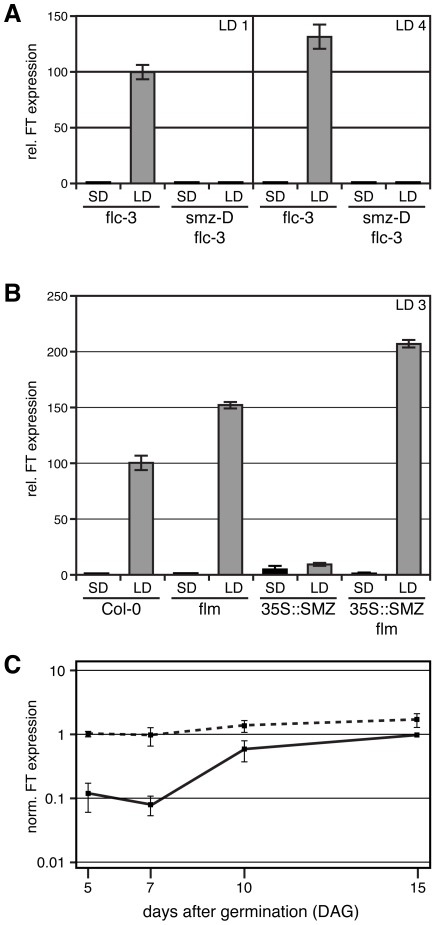
SMZ represses *FT* transcription. The effect of SMZ overexpression on *FT* induction was analyzed by quantitative real-time PCR in wild type, as well as in *flc-3* and *flm-3* mutants. (A) *smz-D* prevents the induction of *FT* by LD in *flc-3* 1 and 4 d after plants were shifted to inductive LD conditions. (B) *35S::SMZ* prevents induction of *FT* in Col-0 after three inductive LD. *FT* expression is restored in a *flm-3* loss-of-function background. Plants were initially grown under noninductive SD conditions, and synchronous flowering was induced by shifting plants to inductive LD. (C) *FT* is precociously expressed in LD-grown *toe1 toe2 smz snz* (dashed line) when compared to wild type (solid line). Plant tissue for RNA extraction was collected at the peak of *FT* expression shortly before the end of the day (ZT = 15 in a 16-h LD). Error bars indicate standard deviation of triplicate measurements.

As expected, *FT* mRNA was not detectable in noninductive SD conditions in *flc-3* mutants, which served as a background for this experiment, irrespective of the presence or absence of *smz-D* (Figure 5A). *FT* transcription was rapidly and strongly induced in *flc-3* 1 d after plants were shifted to inductive LD conditions (Figure 5A). Levels of *FT* mRNA increased even further after exposure to four consecutive LD. In contrast, *smz-D flc-3* plants completely failed to induce *FT*, even after 4 LD. These results indicate that the late flowering observed in *smz-D* is largely caused by the inability of *smz-D* plants to induce *FT* even under inductive LD. Furthermore, the ability of SMZ to repress *FT* did not depend on the presence of a functional *FLC* allele, as already suggested by genetic analyses (Figure 2D).

Genetic analyses had demonstrated that, in contrast to FLC, FLM is strictly required for SMZ to repress flowering. We therefore tested *FT* expression in a *flm* mutant and compared it to that in a *flm 35S::SMZ* line (Figure 5B). As expected, *FT* was readily induced in *flm* after 3 d of inductive LD. *FT* levels were actually higher in *flm* than in Col-0 wild-type control plants, suggesting that FLM is normally involved in *FT* repression. In *35S::SMZ* plants, however, *FT* induction was strongly attenuated, and *FT* levels reached only 8% of those observed in Col-0. In contrast, *FT* was strongly expressed in a *35S::SMZ flm* line, indicating that SMZ requires functional FLM in order to suppress *FT* induction. This is in perfect agreement with our genetic analyses, which had shown that a mutation in *FLM* completely suppresses the late flowering of *SMZ* overexpression.

To test whether SMZ normally represses *FT*, we analyzed its expression in the *toe1 toe2 smz snz* quadruple mutant. We found that *FT* is expressed at high levels in this mutant background when compared to wild-type control plants throughout the first 2 wk of development (Figure 5C). This supports the idea that SMZ, together with the other AP2 family members, represses flowering by regulating *FT* expression.

### Effects of SMZ on Leaf and Shoot Meristem Transcriptome

**Figure 6:**
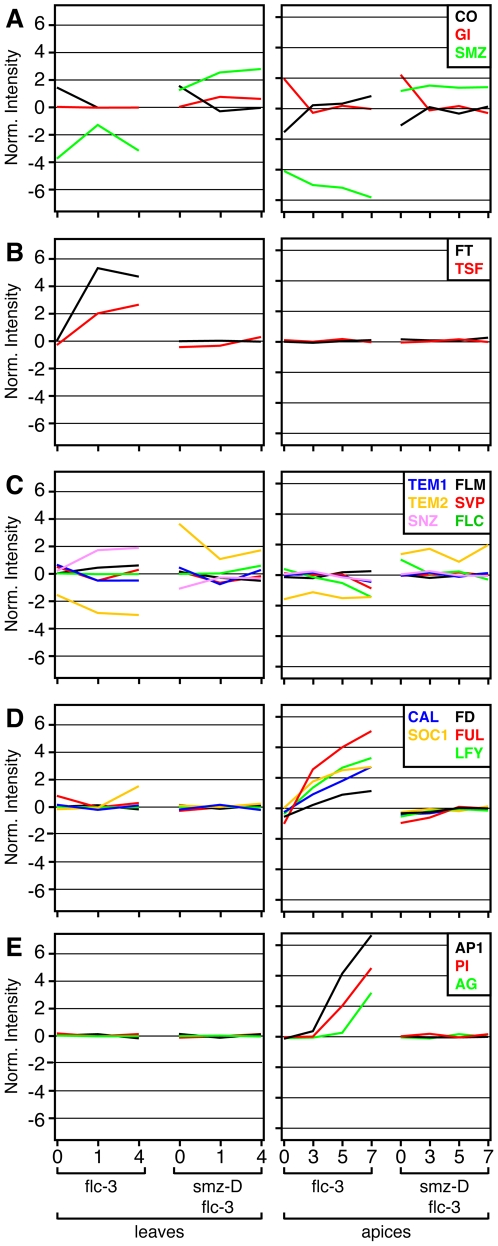
Effects of SMZ overexpression on leaf and meristem transcriptome. Microarray analysis in leaves (left) and at the shoot apical meristem (right) in *flc-3* and *smz-D flc-3*. Changes in gene expression in response to inductive photoperiod were determined 0, 1, and 4 d after the shift to LD in leaves and 0, 3, 5, and 7 d after the shift to LD at the shoot apex. Median normalized expression intensities are shown (log2). *x*-axis: days after shift to inductive LD.

To determine the effects of SMZ overexpression on the transcriptome in greater detail, we performed a microarray analysis in leaves and at the shoot meristem of *flc-3* and *smz-D flc-3* plants. *SMZ* was significantly (RankProducts, percentage false positives [pfp]<0.01) up-regulated in *smz-D* plants at all time points in both tissues investigated (Figure 6A). In contrast, expression of *GIGANTEA* (*GI*) and *CO*, which both act upstream of *FT* in the photoperiod pathway, remained unchanged in *smz-D* plants (Figure 6A). Furthermore, the diurnal expression normally observed of *CO* and *GI* was unaltered (Figure S3), indicating that SMZ is not regulating flowering by modulating expression of these genes. Analysis of *FT* expression by qRT-PCR had revealed that *FT* expression is strongly attenuated in *smz-D* (Figure 5A). In agreement with this, we found that *FT* was significantly (pfp<0.01) induced in *flc-3* leaves 1 and 3 d after plants were transferred to inductive LD but that *FT* induction in leaves was completely blocked by *smz-D* (Figure 6B). *FT* mRNA was not detectable at the shoot meristem at any time point in all samples.

Interestingly, *TWIN SISTER OF FT* (*TSF*) followed the expression of *FT* in that it was substantially up-regulated in leaves of *flc-3* plants, but was not induced at any other time point in any tissues investigated (Figure 6B). This supports the idea that FT and TSF act partially redundantly in promoting the floral transition. Statistical analysis revealed that there was only one other transcript besides *FT* that was significantly (pfp<0.01) induced in leaves of *flc-3* plants in response to inductive photoperiod 1 and 3 d after shift to LD that was not also up-regulated in *smz-D*. This gene encodes a β-amylase (BMY1; At4g15210) and has not previously been implicated in the regulation of flowering.

Taken together, these results indicate that *FT*, and to a lesser extent *TSF*, are major targets of SMZ in leaves. Such a repression of *FT* could either be due to a direct effect of SMZ on the *FT* locus or through the activation of other floral repressors. To investigate the possibility that SMZ is acting indirectly on *FT* by activating transcription of another floral repressor, we examined our *smz-D* microarray data. Many of the known repressors of flowering encode MADS-domain transcription factors, but neither *FLC*, *FLM*, nor *SVP* RNA was induced in *smz-D flc-3*, when compared to *flc-3* (Figure 6C). This is of particular importance, as both FLC and SVP have been shown to bind to the *FT* locus. These results indicate that the delay in flowering observed in *smz-D* plants is not simply caused by *FLC* and/or *SVP* activation. It is, however, possible that SMZ activates or stabilizes FLC and/or SVP protein by an unknown mechanism.

Recently, two AP2-domain transcription factors, TEM1 and TEM2, have also been shown to regulate *FT*. In fact, direct binding of TEM1 to a regulatory region of *FT* has been demonstrated [31]. Although *TEM1* expression is unaltered in both the leaves and at the shoot meristem of *smz-D* plants, *TEM2* was found to be significantly (pfp<0.01) up-regulated in leaves and shoot meristem samples of *smz-D* at all time points (Figure 6C). This suggests that at least part of the effect of SMZ on flowering may be mediated by TEM2.

Interestingly, expression of *SNZ*, the closest paralog of *SMZ*, is significantly (pfp<0.05) down-regulated in leaves of *smz-D flc-3* 1 and 4 d after plants were shifted to LD (Figure 6C). Indeed, even before the shift (day 0), a substantial repression of *SNZ* can be observed. Similarly, levels of *AP2*, *TOE1*, and *TOE3* mRNA are reduced in SMZ overexpressing lines, although to a lesser extent, suggesting widespread feedback regulation among the miR172 target genes (Figure S4).

Positive regulators of flowering such as *FUL*, *LFY*, *CAL*, and *SOC1* were all induced significantly (pfp<0.05) at the apex of *flc-3* plants after the shift to LD (Figure 6D). Similarly, *FD*, which physically and genetically interacts with FT, was substantially (but not significantly) induced at the meristem (Figure 6D). Neither of these genes was, however, induced in *smz-D flc-3*, indicating that high levels of SMZ are sufficient to completely block the transition to flowering at the shoot apex. In agreement with this finding, homeotic genes such as *AP1*, *PI*, and *AG* are also not induced in *smz-D flc-3*, but are readily detectable at the meristem of *flc-3* (Figure 6E).

### Genome-Wide Identification of SMZ Target Genes

**Figure 7:**
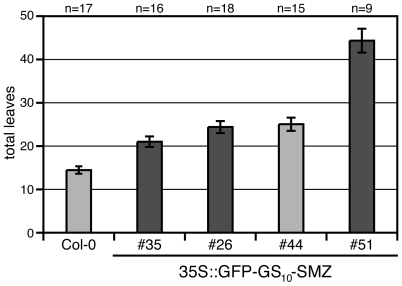
SMZ tagged with GFP remains functional and represses flowering. Transgenic lines constitutively expressing SMZ tagged at the N-terminus with eGFP are late flowering. Flowering times of selected *35S::GFP-SMZ* T2 lines. Error bar indicates 2× SEM.

To determine whether SMZ acts as a regulator of transcription, we established lines that express SMZ in fusion with an N-terminal GFP tag and drove them in the leaves by the 35S promoter. To sustain SMZ function, it was necessary to place a flexible linker consisting of ten Gly-Ser pairs between the GFP and SMZ. Among the T1 lines, several late-flowering individuals were recovered. Late flowering in these lines was confirmed in the T2 generation (Figure 7), indicating that functional GFP∶SMZ protein persisted in these lines at high levels. As expected for a putative transcription factor, the GFP signal was predominately nuclear localized in GFP∶SMZ plants even though signal intensity was rather low when compared to control plants expressing a nuclear-localized 3xVENUS YFP (Figure S5).

The nuclear localization of the GFP fusion protein enabled us to perform chromatin immunoprecipitation on whole-genome tiling arrays (ChIP-chip) in order to identify regions in the *Arabidopsis* genome bound by SMZ. In total, 434 regions in the genome exhibited statistically significant enrichment to GFP∶SMZ at a false discovery rate (FDR) of 5% or less when compared to a line expressing a nuclear-localized YFP (Dataset S5). Of these 434 peak regions, only 33 were not directly associated with genes (±2.5 kb of the coding sequence [CDS]), whereas the great majority (401 peaks) fell within 2.5 kb of annotated genes. The latter were associated with 395 unique *Arabidopsis* gene models with six annotated loci having two peaks of significant binding.

We observed several interesting enrichments for particular gene ontology (GO) categories among the 307 of 395 genes for which assignments exist. Indeed, genes associated with flower development (GO:0009908) were significantly overrepresented at a FDR *p*<0.0005 among the list of potential SMZ target genes, demonstrating a functional specificity to binding and a nonrandom distribution of the peaks identified by ChIP-chip. The second biological process found to be overrepresented at a FDR *p*<0.0005 comprises genes involved in “response to stimuli” (GO:0050896), in particular to water (GO:0009415, GO:0009414) and jasmonic acid (GO:0009753).

**Figure 8:**
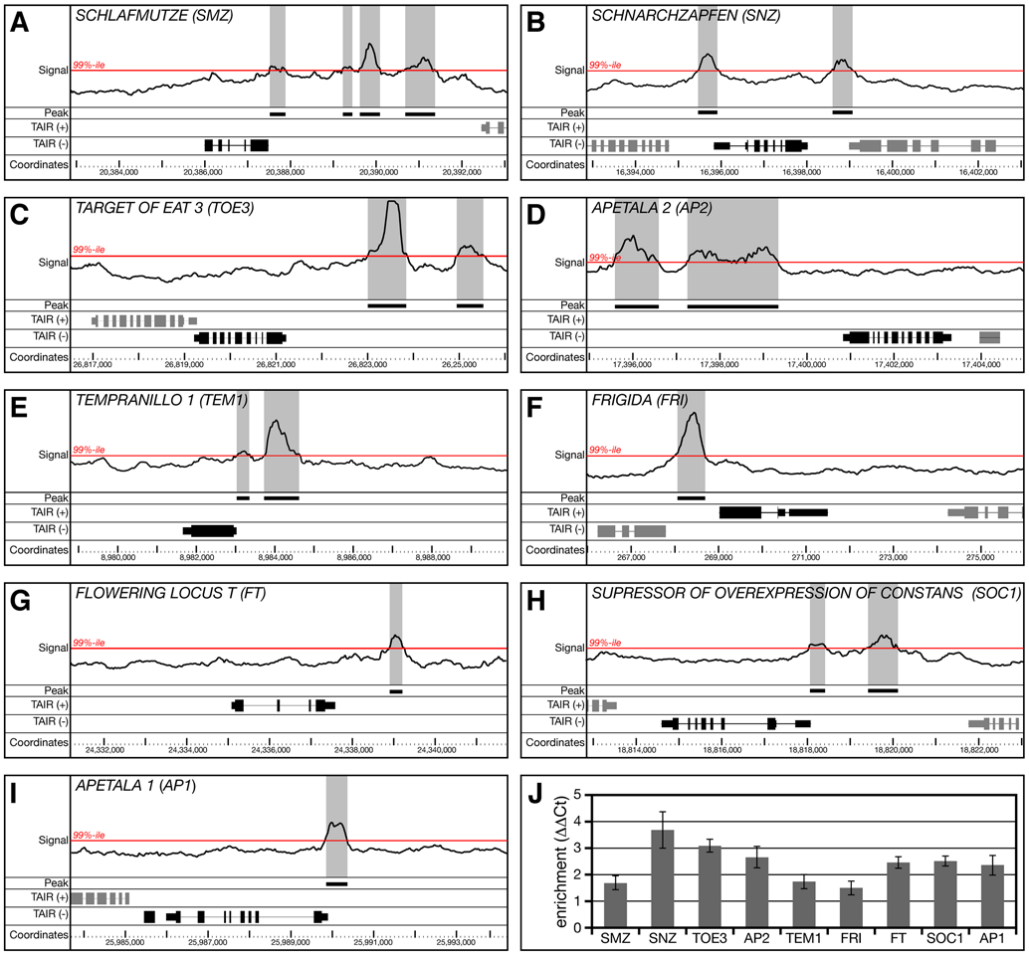
Identification of SMZ targets by ChIP-chip. (A–D) SMZ binds to the genomic regions of (A) *SMZ*, (B) *SNZ*, (C) *TOE3*, and (D) *AP2*, suggesting extensive feedback regulation among the miR172 target genes. (E and F) SMZ binds to the promoters of the floral repressors (E) *TEM1* and (F) *FRI*. (G–I) The floral integrator and flower development genes (G) *FT*, (H) *SOC1*, and (I) *AP1* are directly bound by SMZ. (J) Binding of SMZ to the regulatory regions identified by ChIP-chip was confirmed by quantitative PCR. Bound regions (peaks) are highlighted in grey. All peaks fall within the top 1% of enriched regions (99th percentile), and at least one peak per gene is statistically significant at a FDR <5%. For a complete list of all 434 regions bound by SMZ at a FDR <5%, see Dataset S5.

Among the genes bound by SMZ were many known regulators of flowering, suggesting that the effects of SMZ on flowering time are rather direct. Most notably, the second most strongly enriched locus in the entire genome analysis was located less than 2 kb upstream of the transcription start site of the miR172 target gene *TOE3* (Figure 8C). Binding at the *TOE3* locus was highly statistically significant (FDR<0.000001). Closer inspection of the list of high-confidence SMZ targets (FDR<5%) revealed that three other miR172 targets were also significantly bound. Among them were *SMZ* itself, its paralog *SNZ*, and *AP2* (Figure 8A, 8B, and 8D). In fact, *SMZ* was one of only six loci genome-wide that was the closest locus to two peaks of high-confidence binding. No high-confidence binding by SMZ was detected to the last two members of the clade of miR172 targets, *TOE1* and *TOE2*. The finding that four out of six miR172 targets were significantly bound by SMZ is more than expected by chance (Fisher exact test; *p*<0.0001). It should be noted that the expression levels of *SNZ*, *AP2*, and *TOE3* were reduced in leaves of *smz-D* plants as measured by microarrays (Figure 6C and Figure S4). Taken together, these results strongly suggest a complex negative regulatory feedback mechanism among the miR172 targets. In addition, two other known repressors of flowering, *TEM1* and *FRI*, were also bound by SMZ (Figure 8E and 8F).

Most interestingly, *FT* was also among the genes that showed significant binding by SMZ approximately 1.5 kb downstream of the *FT* CDS (Figure 8G). Assuming that SMZ acts as a transcriptional repressor, the binding of SMZ to the *FT* locus readily explains the failure of *smz-D* and *35S::SMZ* plants to induce *FT* (Figure 5A and 5B) and the high levels of *FT* expression in the *toe1 toe2 smz snz* quadruple mutant (Figure 5C). The regulatory landscape around the FT locus appears to be rather complex, with TEM1 binding to the 5′UTR, FLC binding to the first intron, and finally, SMZ binding downstream of the coding region [25],[29],[31]. Further, the effect of high SMZ levels on flower development is most likely not only due to a repression of *FT*, but also to SMZ repressing other flowering time regulators as well. This idea is supported by the finding that, besides *FT*, the floral integrator and flower development genes *SOC1* and *AP1* were also identified by ChIP-chip as high-confidence (FDR<5%) targets of SMZ (Figure 8H and 8I). In each of these cases, binding occurred directly upstream of the transcription start site, suggesting strongly that expression of these genes is under direct negative regulation by SMZ. Similar to what we had observed for *FT*, *smz-D* plants did not induce *SOC1* or *AP1* either in leaves or at the shoot meristem when shifted from SD to inductive LD (Figure 6D and 6E). Enrichment of loci identified by ChIP-chip was confirmed by quantitative PCR for all genes discussed (Figure 8J).

To test whether SMZ and its paralogs affect expression of the genes bound by SMZ, we analyzed their expression by quantitative PCR in the *toe1 toe2 smz snz* quadruple mutant. As already described for *FT* (Figure 5C), *AP1* and *SOC1* were strongly induced in the quadruple mutant (Figure S6). So were *LFY* and *FUL* (Figure S6), which are not or only weakly bound by SMZ. The latter most likely is due to indirect activation of these genes. The expression of *AP2* and *TOE3* was also increased. This is in agreement with the proposed negative feedback regulation among the miR172 target genes. In contrast, *FRI* was only marginally up-regulated (Figure S6). However, the *FRI* allele of the Col-0 accession, is recessive and essentially nonfunctional, making it hard to interpret this result [39].

Our results strongly support the notion that SMZ, and by extension the other miR172 targets as well, act as direct repressors of the transition to flowering. Consistent evidence from both gene expression and ChIP-chip experiments suggests that SMZ directly represses the transcription of a range of known flowering time genes and that the delay in flowering time caused by high levels of SMZ is most likely a result of repression of a number of flowering time regulators in both the leaves and the shoot meristem.

## Discussion

**Figure 9:**
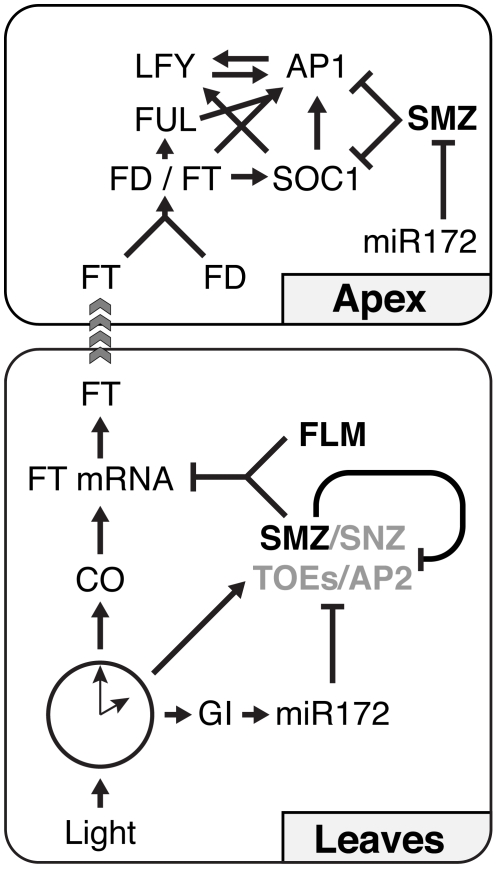
Genetic Interactions governing photoperiodic flowering. Photoperiod is perceived in leaves and entrains the circadian clock. *CONSTANS* (*CO*) is a major output of the clock in terms of regulating flowering. CO is activating expression of *FLOWERING LOCUS T* (*FT*) specifically under LD conditions. This activation of *FT* is counteracted by floral repressors such as SMZ, which itself is negatively regulated by miR172. SMZ is also repressing related genes in a negative feedback loop. At the shoot apex, SMZ binds to regulators sequences of *APETALA1* (*AP1*) and *SUPRESSOR OF CONSTANS OVEREXPRESSION* (*SOC1*), two other known regulators of flowering time and floral development.

To ensure reproductive success, plants have evolved a complex regulatory network that integrates various endogenous and environmental factors to ensure that flowering occurs when conditions are most favorable. Many of the key regulators that control flowering time have been identified and the majority of them are putative transcription factors. Extensive epigenetic regulation of several key regulators of flowering complicates the situation even further [40]. Based on genetic analyses, pathways that control the transition to flowering have been defined, but the details of how this transcription factor network functions at a molecular level is poorly understood. Here, we have combined genetic analysis with high-throughput microarray technologies to understand in detail how the AP2-like transcription factor SMZ represses flowering. A model summarizing our findings regarding the genetic interactions of *SMZ* and its position in the network regulating flowering in response to photoperiod is represented in Figure 9.

SMZ is predominately expressed in young leaves, suggesting that this is the tissue where it normally functions [35],[41]. Leaves play a crucial role in the perception of day length, and it has recently been demonstrated that the information to induce flowering can be conveyed from the leaves to the shoot apex via transport of the FT protein [18]–[23]. The regulation of *FT* expression is therefore of the utmost importance for a plant to ensure the correct timing of flowering. Plants achieve this control by a combined effect of activators of *FT* expression, such as CO, and repressors, such as FLC, FLM, SVP, and the TEM proteins, some of which have been shown to directly bind to regulatory regions of the *FT* locus [25],[29],[31]. Expression of SMZ from a leaf-specific promoter recapitulated the late-flowering phenotype of constitutive SMZ overexpression, indicating that presence of SMZ in the vasculature was sufficient to repress flowering. Molecular analyses indicate that SMZ directly contributes to the regulation of *FT* in leaves. The evidence for this is 2-fold: first, plants expressing SMZ at high levels fail to induce *FT* in response to LD, and second, SMZ binds directly to the *FT* locus. Taken together, these results strongly indicate that SMZ acts as a floral repressor and that *FT* is a major transcriptional target of SMZ in leaves.

Whether FT constitutes the sole mobile signal that conveys the instruction to flower from leaves to the apex is still an open question. Several other classes of molecules have been implicated as long-distance flowering signals in various plant species [42],[43]. Carbohydrates in general, and sucrose in particular, have been suggested to play a role in the induction of flowering in *Arabidopsis* [44],[45]. The mechanism by which sugars affect flowering is not entirely clear, but the finding that SMZ binds to and represses *BMY1*, which encodes a cytosolic β-amylase, may provide new insights into this issue.

Misexpression of miR172-resistant SMZ from a meristem-specific promoter had a marked effect on flower development, and *FD::rSMZ* plants phenotypically resembled *ft lfy*, *fd lfy*, or *lfy ap1* double mutants [16],[17],[46]. Although we did not detect high-confidence binding of SMZ to *LFY*, strong, significant binding was observed to *SOC1* and *AP1*, so it is possible that the reduced abundance of these factors at the meristem may be at least partly responsible for the observed phenotype. Along these lines, it has recently been reported that a *soc1 ful* double mutant reverts to a vegetative state after flowering had been induced, resulting in a perennial growth habit of the double mutant and demonstrating the importance of these genes in robustly inducing flowering [47].

Furthermore, SMZ does not act alone in repressing flowering but instead redundantly with related proteins, all of which are miR172 target genes. It has previously been shown that miR172 overexpression did not dramatically alter the mRNA levels of its targets. This has been interpreted as evidence for translational repression being more important than mRNA cleavage [36]. Later, however, it was shown that at least one of these genes, *AP2*, can repress its own transcription, demonstrating that a negative feedback very much confounds this conclusion [48]. It was, however, not clear whether this repression was direct or indirect. Also, it was unclear just how widespread this negative feedback regulation among the miR172 targets really was. Our genome-wide ChIP binding and gene expression studies indicate that SMZ is not only binding to its own genomic region, but regulates at least three other family members as well, demonstrating that the negative feedback is direct and common among the miR172 targets.

The function of SMZ appears to strictly depend on the presence of the MADS-domain transcription factor FLM. The mechanistic details of this interaction remain unknown, but one can imagine several possible scenarios. SMZ might directly interact with FLM protein to form a repressor complex. However, at least when tested in yeast, we did not find any indication for direct interaction between SMZ and FLM (unpublished data). An alternative would be that FLM needs to be present at target loci in order to facilitate either SMZ binding or activity, but without physical interaction between the two. The dependence of SMZ on FLM seems to be rather specific, as inactivation of the MADS repressors FLC nor SVP, both of which have been shown to directly repress *FT*, does not prevent SMZ function. It has been suggested that FLM and SVP genetically act as partners in repressing flowering time [28]. However, at least in the case of repressing SMZ activity, FLM and SVP functions are clearly separate and not interchangeable.

In addition to the genes discussed so far, *TEM1*, a known repressor of flowering, was also bound by SMZ (Figure 8E). How binding of *TEM1* by SMZ could possibly regulate flowering is currently unclear. It should be noted that the expression levels of *TEM1* were not changed in leaves and at the meristem by *smz-D* (Figure 6C). In contrast, the expression levels of *TEM2*, the closest paralog of *TEM1*, were significantly up-regulated in leaves and meristem samples (Figure 6C). One may hypothesize that, similar to what we have observed among the miR172 targets, control of *TEM1* and *TEM2* expression also involves a regulatory feedback mechanism.

Regardless of the precise mechanisms that control *TEM* expression, the increased *TEM2* levels in *smz-D* very likely contribute to the repression of *FT*. Finally, *FRIGIDA* (*FRI*) was also identified among the genes most strongly bound by SMZ (Figure 8F). FRI is a potent activator of *FLC*, and together these two genes are to a large extent responsible for the winter-annual behavior of certain *Arabidopsis* accessions [24],[39],[49]. Col-0 carries a recessive *FRI* allele, and it is therefore unlikely that the binding of SMZ to the *FRI* promoter is responsible for the delay in flowering we observe in *smz-D*. However, SMZ and the other miR172 targets could very well contribute to the control of *FRI* expression in late-flowering accessions that carry a functional *FRI* allele. This would provide *Arabidopsis* with a way to regulate *FLC* levels by modulating FRI expression. To test such a scenario, one would need to analyze the effect of gain- and loss of function of miR172 targets on flowering time and especially *FLC* expression levels in a *FRI* dominant background.

Our results indicate that the miR172/SMZ module functions as a rheostat in flowering time by SMZ binding to several genes encoding miR172 targets and other flowering time regulators. The importance of this regulatory module is highlighted by the finding that overexpression of miR172 strongly accelerates flowering [33],[36], whereas constitutive expression of *SMZ* (or *AP2*, *TOE1*, or *TOE2*) has the opposite effect [33],[34],[36]. In nature, to tightly control flowering time, *Arabidopsis* must achieve a careful balance between miR172 and its targets. Negative feedback of SMZ onto the other miR172 targets likely contributes to this regulation. miR172 and its targets are not specific to *Arabidopsis*, but are conserved in other dicotyledonous as well as monocotyledonous plant species, suggesting that these genes play an important role in plant development in general [50]. In maize, for example, miR172 promotes vegetative phase change and onset of reproductive development [51], indicating that the function of the miR172/AP2 module is largely conserved. In addition, miR172 and its target *indeterminate spikelet1* (*ids1*) have been shown to participate in sex determination and meristem cell fate in maize [52]. Thus, our findings about the regulatory module consisting of AP2-like transcription factors and their microRNA will likely be relevant to many other plants.

In summary, we provide evidence for a complex regulatory feedback mechanism among the miR172 target genes that directly controls the expression of FT. In addition, we show that several other known flowering time regulators such as *SOC1* and *AP1* are also directly targeted and repressed by SMZ. The intricate regulatory interactions we uncovered by just looking at just one single factor, SMZ, demonstrate how complex regulation of flowering time at the molecular level is. To fully understand this complex trait, a concerted effort of the flowering time community will be required to systematically study the genes and proteins involved in floral transition on a genome-wide scale.

## Material and Methods

Sequences of oligonucleotide primers used in this work are given in Table S2.

### Plant Material

Wild-type plants were of the Columbia (Col-0) accession. All T-DNA insertion mutants used in this work are in Col-0 accession [53],[54]. *flc-3*, *flm-3*, *svp-31*, *toe1-2*, and *toe2-1* have been described before [24],[30],[33],[55]. Two T-DNA insertion lines for SMZ (*smz-1* and *smz-2*) and one SNZ loss-of-function allele (*snz-1*) were isolated as part of this work (Table S1). *smz-2* was used for genetic analysis throughout this work. Mutant plants were confirmed by PCR-based genotyping.

### Growth Conditions

All plants were grown in growth chambers in a controlled environment (23°C, 65% relative humidity). Plants were raised on soil under a mixture of Cool White and Gro-Lux Wide Spectrum fluorescent lights, with a fluence rate of 125 to 175 µmol m^−2^ s^−1^. All light bulbs were of the same age. Long day (LD) is defined as 16 h light, 8 h dark, and short days (SD) as 8 h light, 16 h dark. For flowering time measurements, plants were randomized with the respective controls, and the flowering time phenotype was determined without prior knowledge of the genotype. All flowering time assays were performed at least twice.

### Cloning

ORFs were amplified using the *Pfu* DNA polymerase (New England Biolabs), cloned into Gateway entry vectors using T4 DNA ligase and subsequently recombined into Gateway-compatible binary vectors suitable for plant transformation. Constructs for constitutive and tissue-specific expression of *SMZ* were obtained by amplification of the *SMZ* ORF using oligonucleotide primers G-3323 and G-5638, cloning the PCR product into pJLSmart, resulting in pJM9, and recombination into Gateway-compatible plant binary vectors, providing promoters for expression in plants, generating pJM34 (*35S::SMZ*), pJM66 (*SUC2::SMZ*), and pJM50 (*FD::SMZ*). To generate the *miRNA172*-resistant form of *SMZ* (*rSMZ*), synonymous mutations were introduced into the *miR172* binding site by site-directed mutagenesis using oligonucleotide primers G-2050 and G-2051, resulting in pFK37. Cloning of *rSMZ* into Gateway-compatible entry and destination vectors was as described above, resulting in pJM36, pJM68, and pJM52 (for *35S::rSMZ*, *SUC2::rSMZ*, and *FD::rSMZ*, respectively). To separate SMZ from the GFP tag, a Gly-Ser linker was added to the N-terminus of the *SMZ* ORF in a two-step PCR. In a first PCR, the *SMZ* ORF was amplified using primers G-16615, which replaced the start codon of *SMZ* with 30 bases encoding for five Gly-Ser pairs, and G-16616. In a second step, the Gly-Ser linker was extended to its final length of 60 bases, encoding for ten Gly-Ser pairs, using G-18665 and G-16616. The resulting PCR product was cloned into the SmaI site of the pJLSmart Gateway-compatible entry vector by blunt end ligation (pFK478). The *GS_10_∶SMZ* ORF was subsequently recombined into a pGREEN-IIS based Gateway-compatible destination vector (pFK247), which provided 35S promoter for expression in plants and an in-frame fusion with an N-terminal eGFP, resulting in pFK480. For the genomic SMZ∶GUS reporter, a 10.6-kb EcoRV-SacI, including the whole SMZ 5′ and 3′ regions, was cut from BAC T15C9 and cloned into the pGREEN-IIS plant binary vector. The GUS ORF, including stop codon, was amplified by PCR using primers G-8237 and G-8238, introducing AgeI sites in the process. The GUS ORF was cloned in frame with the SMZ start codon in an AgeI site present in the first exon of SMZ. All sequences amplified by PCR were confirmed by sequencing. All enzymes used were purchased from Fermentas unless otherwise indicated. Complete sequences of constructs used are available on request. For sequences of the primers used to amplify ORFs, see Table S1.

### Plant Transformations

For plant transformation, constructs were transformed into *Agrobacterium tumefaciens* strain ASE by electroporation. *Arabidopsis* plants of the Col-0 accession were transformed by the floral-dip procedure [56]. Transgenic plants were selected with 0.1% glufosinate (BASTA) on soil or 50 µg/ml kanamycin on plates. At least 20 T1 plants were analyzed for each construct.

### Total RNA Extraction and Quantitative Real-Time PCR

Total RNA was extracted from plant tissue using either the Plant RNeasy kit (Qiagen) or Trizol reagent (Invitrogen) according to the manufacturer's instructions. 2 µg of total RNA was DNase I-treated and single-stranded cDNA was synthesized using oligo(dT) and the RevertAid First Strand cDNA Synthesis Kit (Fermentas). Quantitative real-time PCR was performed on an Opticon Continuous Fluorescence Detection System (MJR) using the Platinum SYBR Green qPCR Supermix-UDG (Invitrogen). Gene expression was calculated relative to β-Tubulin using the ΔΔCT method. Results are reported for triplicate measurements of one of several biological replicates. For each genotype and replicate, a minimum of 10 seedlings was pooled for RNA extraction. Oligonucleotide primers used for qRT PCR are listed in Table S1.

### Microarray Expression Analysis

For the analysis of the leaf transcriptome in *Arabidopsis flc-3* and *smz-D flc-3*, plants were grown under SD conditions for 14 days and shifted to LD to induce flowering synchronously. Rosette leaves one to three from 10 plants were collected zero, 1 and 4 d after the plants were shifted to LD in duplicate, and total RNA was extracted using Qiagen Plant RNeasy columns (Qiagen). Biotinylated antisense RNA was prepared from 1 µg of total RNA using the MessageAmp II-Biotin Enhanced Kit (Ambion) according to the manufacturer's instructions. A total of 13.5 µg of fragmented amplified RNA (aRNA) was hybridized to an *Arabidopsis* ATH1-121501 gene expression array (Affymetrix). Arrays were washed and stained on a GeneChip Fluidics Station 450 (Affymetrix) and scanned on an Affymetrix GeneChip scanner GS300 7G. Analysis of the shoot meristem transcriptome was carried out as described above except that plants were grown for 25 days under SD before transfer to LD. RNA from shoot apices was isolated as described [34]. For visualization, normalized expression estimates were obtained by directly importing .CEL files into GeneSpring 10 using gcRMA (Agilent Technologies) and baseline transformation as a normalization routine. All microarray data are freely available from the ArrayExpress database (http://www.ebi.ac.uk/arrayexpress; accession numbers: E-MEXP-2040 (leaf samples) and E-MEXP-2041 (apices)). Lists of statistically significantly expressed genes (Datasets S1, S2, S3, and S4) were calculated for pairwise comparisons between time points within a given genotype or between genotypes at a given time point using RankProducts (version 2.6.0) implemented in R (version 2.4.0; GUI 1.17) on gcRMA (version 2.6.0) normalized expression estimates [57],[58].

### Crosslinking, Chromatin Isolation, and ChIP-Chip

The entire ChIP-chip experiment from sonication through array analysis was performed on technical duplicate samples from both 35S::NLS-3xVenusGFP and 35S::GFP-SMZ seedlings and then repeated on biological replicate samples. Briefly, seedlings grown for 9 LD were fixed at the end of the day as described previously [59]. Frozen tissue was ground, filtered three times through Miracloth (Calibrochem), and washed as described previously thorough buffers M1, M2, and M3 [59]. Nuclear pellets were resuspended in sonic buffer as described (1 mM PEFA BLOC SC [Roche Diagnostics] was substituted for PMSF), split into duplicate samples, and sonicated with a Branson sonifier at continuous pulse (output level 3) for eight rounds of 2×6 s and allowed to cool on ice between rounds. Immunoprecipitation (IP) reactions were performed by incubating chromatin with 2.5 µl of anti-rabbit GFP antibody (ab290, Abcam) overnight at 4°C, as described [59]. The immunoprotein–chromatin complexes were captured by incubating with protein A-agarose beads (Santa Cruz Biotechnology), followed by consecutive washes in IP buffer and then elution as described [59]. Immunoprotein-DNA was then incubated consecutively in RNase A/T1 mix (Fermentas) and Proteinase K (Roche Diagnostics) as described, after which DNA was purified using Minelute columns (Qiagen) [59]. Recovered DNA was amplified using the Sigma WGA GenomePlex kit (Sigma-Aldrich), after we performed a comparison to other systems, which showed this protocol gives improved amplification consistency and minimal amplification bias, in accordance with a previous study [60]. One microgram of DNA was fragmented, labeled, and hybridized to Affymetrix Arabidopsis tiling 1.0F arrays (Affymetrix). Chromatin size distribution and fragmentation performance was confirmed on an Agilent Bioanalyzer prior to array hybridization (Agilent Technologies). Regions found to be enriched by ChIP-chip were confirmed by manual ChIP. We performed triplicate qPCR on chromatin samples from 35S::SMZ-GFP and 35S::GFP-NLS plants. As a negative control, we used a region 3.5 kb away from the FT peak that was not enriched in ChIP-chip analysis.

### Primary Array Analysis

Tiling array data were processed using the CisGenome suite [61]. Briefly, raw .CEL files were quantile normalized and peaks were called using TileMapv2. Analysis was performed in MA mode with window size 5, and only peaks detected with a FDR of better than 0.05 were analyzed. EasyGO was used to do GO-based enrichment analysis [62]. Genome-wide visualization was performed with Affymetrix Integrated Genome Browser after normalization with Affymetrix Tiling Array Software (Affymetrix). All tiling array data are freely available from the ArrayExpress database (http://www.ebi.ac.uk/arrayexpress; accession numbers: E-MEXP-2068).

## Supporting Information

Dataset S1 **Probe set identifiers of differentially expressed genes.** Excel spreadsheet containing ATH1 probe set identifiers of genes differentially expressed in pairwise comparisons (within genotypes and between geneotypes).
(0.12 MB XLS)

Dataset S2 **Genes regulated by SMZ in leaves.** Excel spreadsheet containing lists of ATH1 probe set identifiers differentially expressed (RankProducts; pfp>0.05) between *flc-3* and *smz-D flc-3* in leaves.
(0.05 MB XLS)

Dataset S3 **Genes regulated by SMZ at the shoot apex.** Excel spreadsheet containing lists of ATH1 probe set identifiers differentially expressed (RankProducts; pfp>0.05) between *flc-3* and *smz-D flc-3* in apices.
(0.04 MB XLS)

Dataset S4 **RankProducts results.** Compressed folder containing results from all RankProducts pairwise comparisons (pfp<0.05; 100 permutations) within a genotype (*flc-3*; *smz-D flc-3*) or between genotypes at a given time point for leaves and meristems.
(0.32 MB ZIP)

Dataset S5 **Genomic loci bound by SMZ.** Excel spreadsheet containing information on 434 chromosomal regions that were significantly enriched in 35S::GFP∶SMZ compared to 35S::3xYFP-NLS at a FDR rate of <5%.
(0.24 MB XLS)

Figure S1 **Statistical analysis of distribution of flowering time by rosette, cauline, and total leaf number in** ***miR172*** **loss-of-function lines.**
(0.29 MB TIF)

Figure S2 **Mapping of miR172 cleavage sites in** ***SMZ*** **and** ***SNZ*** **mRNA.**
(0.14 MB TIF)

Figure S3 **Diurnal expression of** ***GI*** **and** ***CO*** **in** ***smz-D*** **.**
(0.55 MB TIF)

Figure S4 **Expression profiles of miR172 target genes in leaves and at the shoot apex.**
(0.12 MB TIF)

Figure S5 **Subcellular localization of GFP∶SMZ fusion protein.**
(1.96 MB TIF)

Figure S6 **Expression of flowering time genes in the** ***toe1 toe2 smz snz*** **quadruple mutant.**
(0.39 MB TIF)

Table S1 **Mutant lines used throughout this work.**
(0.05 MB DOC)

Table S2 **Oligonucleotides used in this work.**
(0.13 MB DOC)

## Abbreviations

ChIP-chip: chromatin immunoprecipitation coupled to hybridization to tiling arrays
FDR: false discovery rate
GO: gene ontology
LD: long day
pfp: percentage false positives
SD: short day
