# Supplementary figures and images for: Repression of Flowering by the miR172 Target SMZ
Source: PLoS Biol. 2009 Jul 7;7(7):e1000148. doi: 10.1371/journal.pbio.1000148 (PMC2701598; doi:10.1371/journal.pbio.1000148)

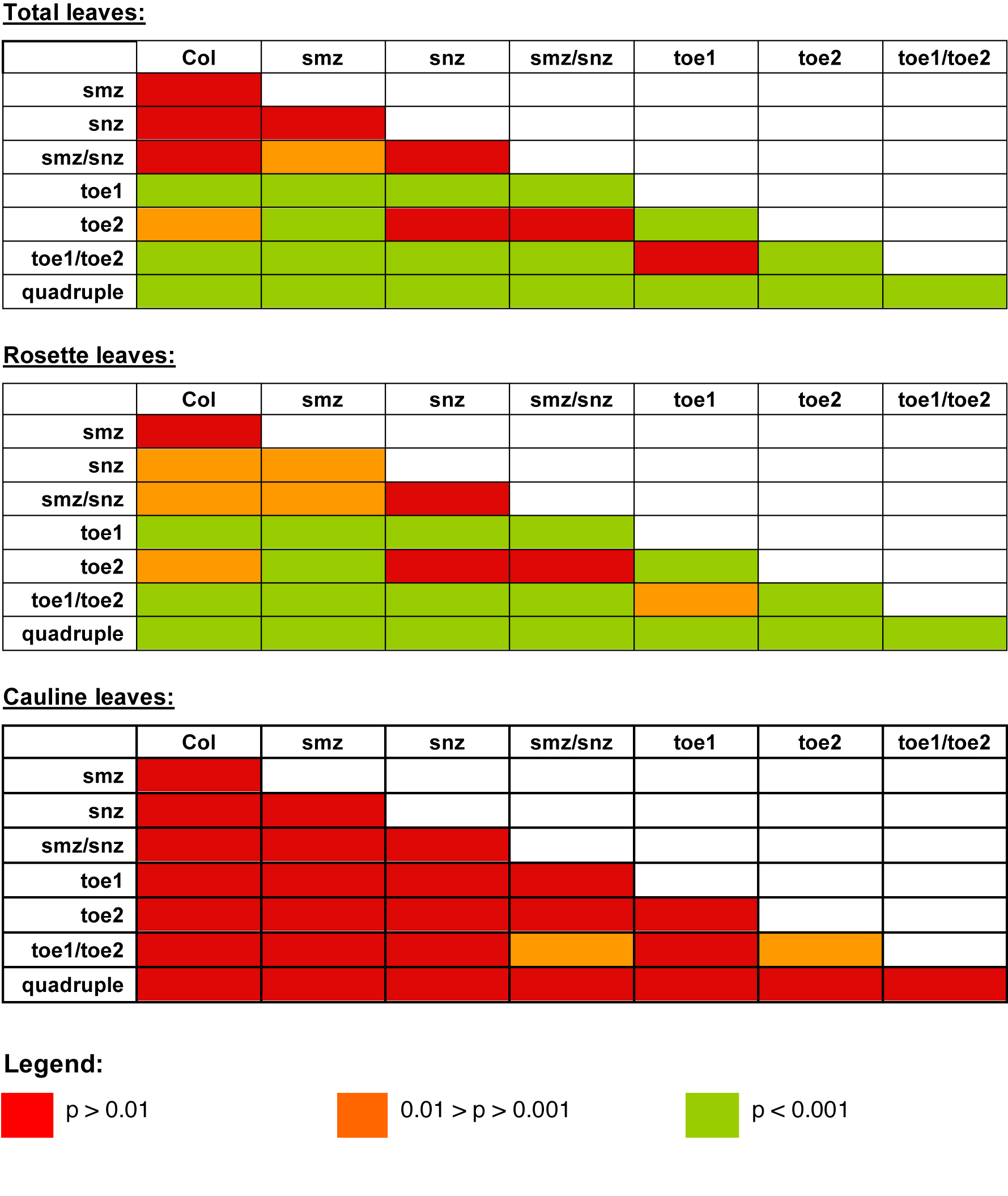

Supplement: Figure S1 — Statistical analysis of distribution of flowering time by rosette, cauline, and total leaf number in miR172 loss-of-function lines. (0.29 MB TIF) [file pbio.1000148.s006.tif]

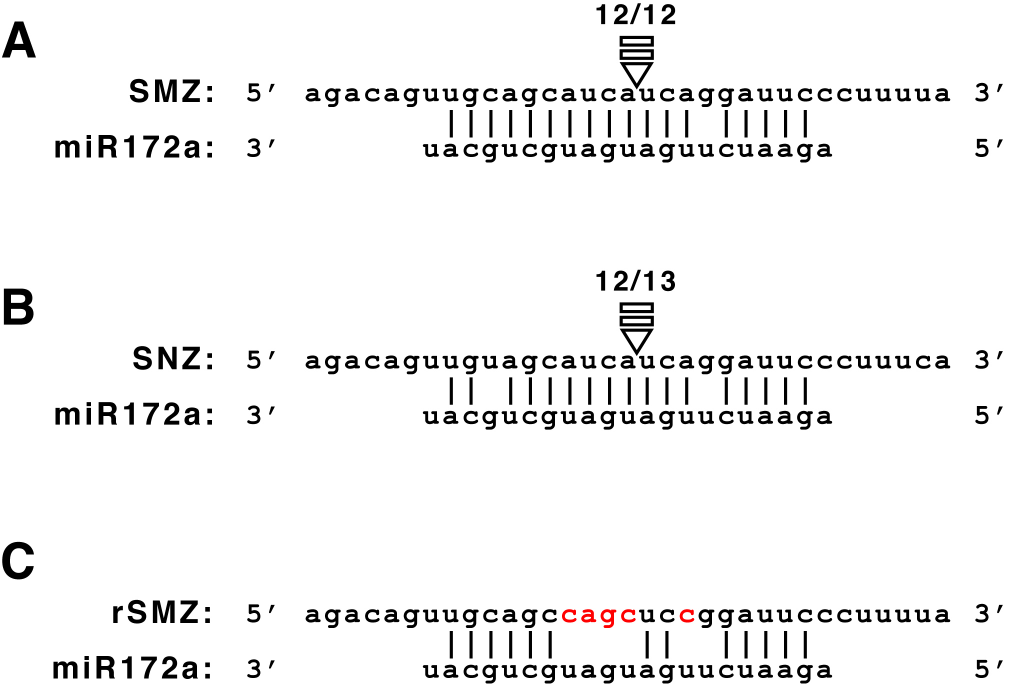

Supplement: Figure S2 — Mapping of miR172 cleavage sites in SMZ and SNZ mRNA. (0.14 MB TIF) [file pbio.1000148.s007.tif]

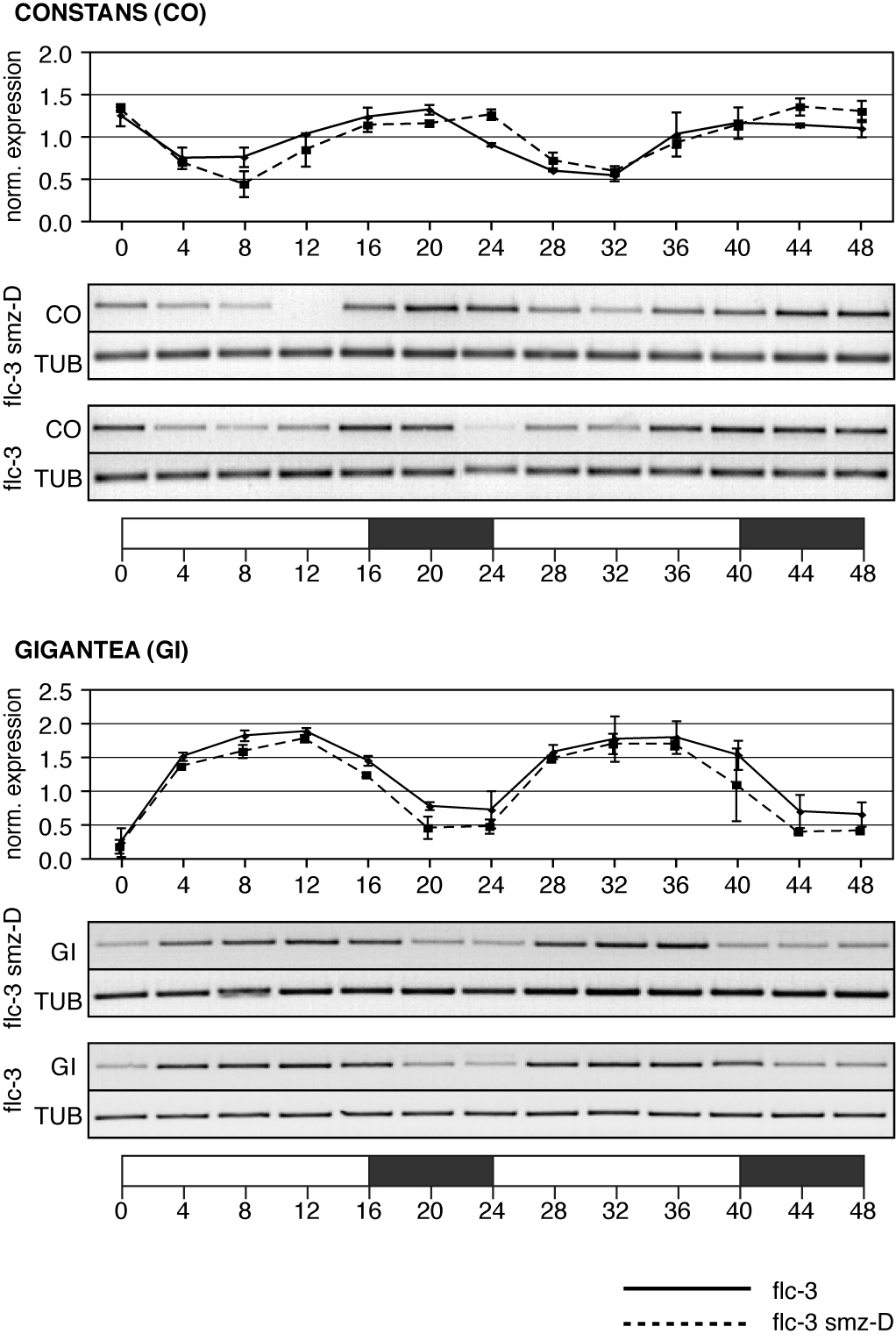

Supplement: Figure S3 — Diurnal expression of GI and CO in smz-D . (0.55 MB TIF) [file pbio.1000148.s008.tif]

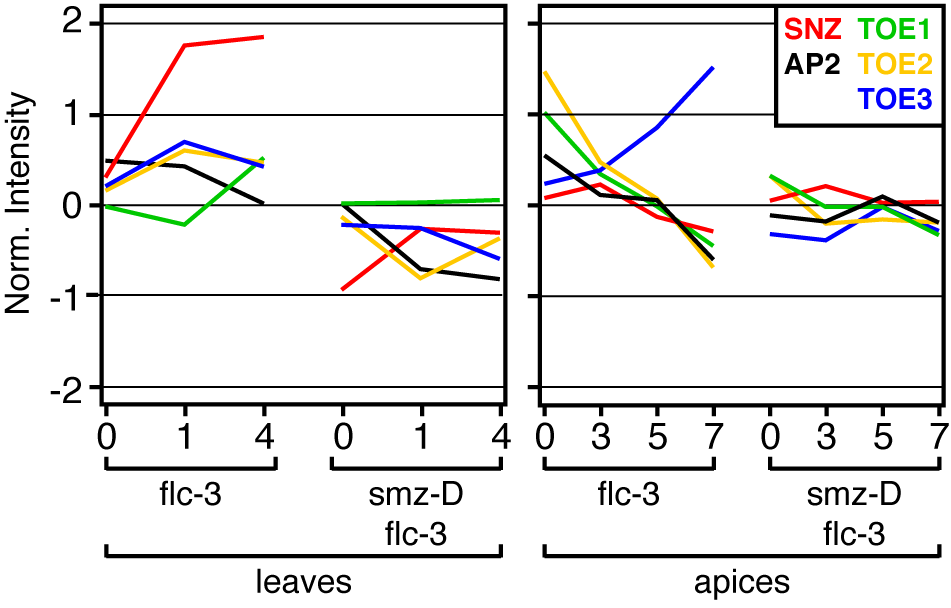

Supplement: Figure S4 — Expression profiles of miR172 target genes in leaves and at the shoot apex. (0.12 MB TIF) [file pbio.1000148.s009.tif]

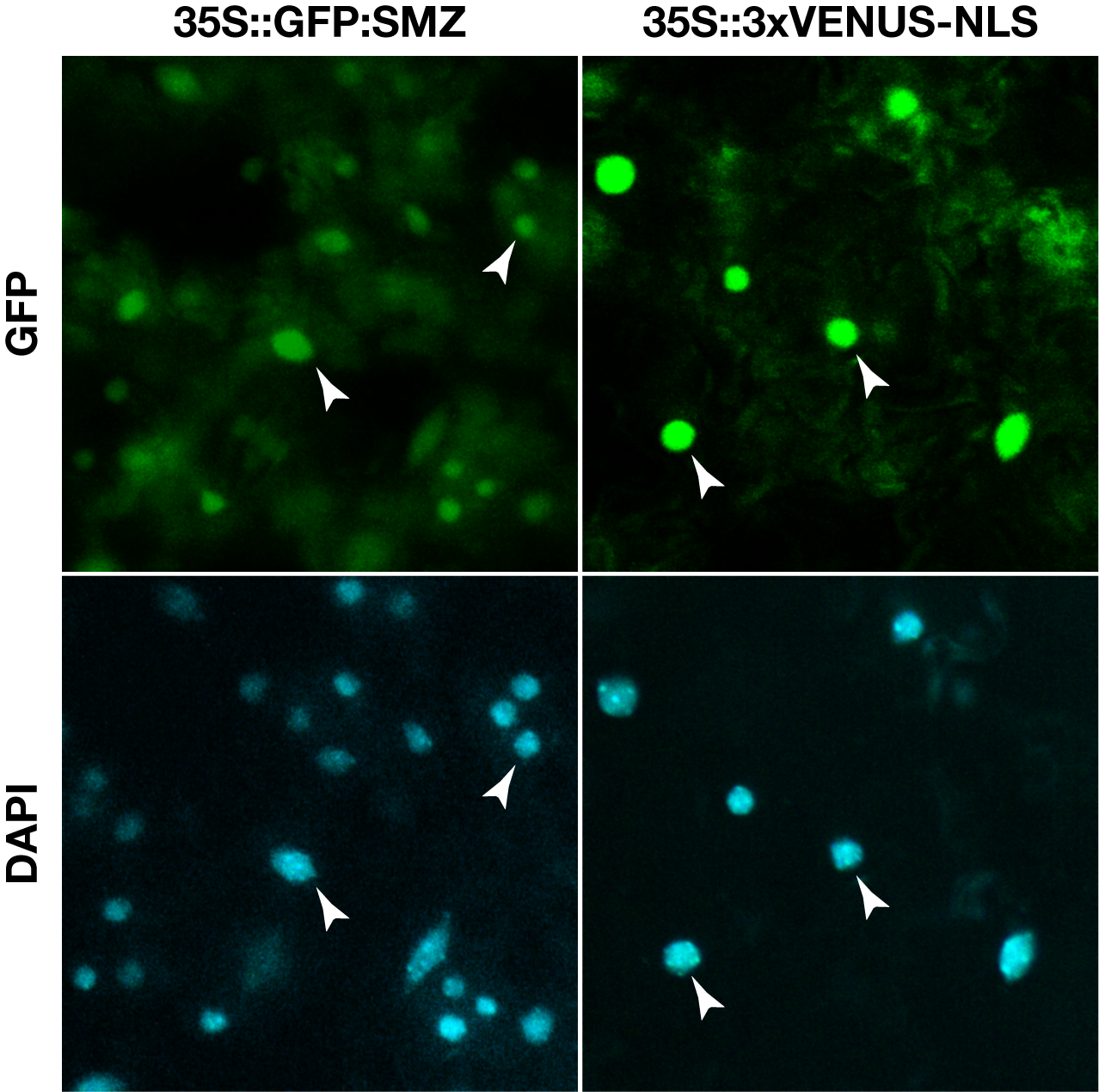

Supplement: Figure S5 — Subcellular localization of GFP∶SMZ fusion protein. (1.96 MB TIF) [file pbio.1000148.s010.tif]

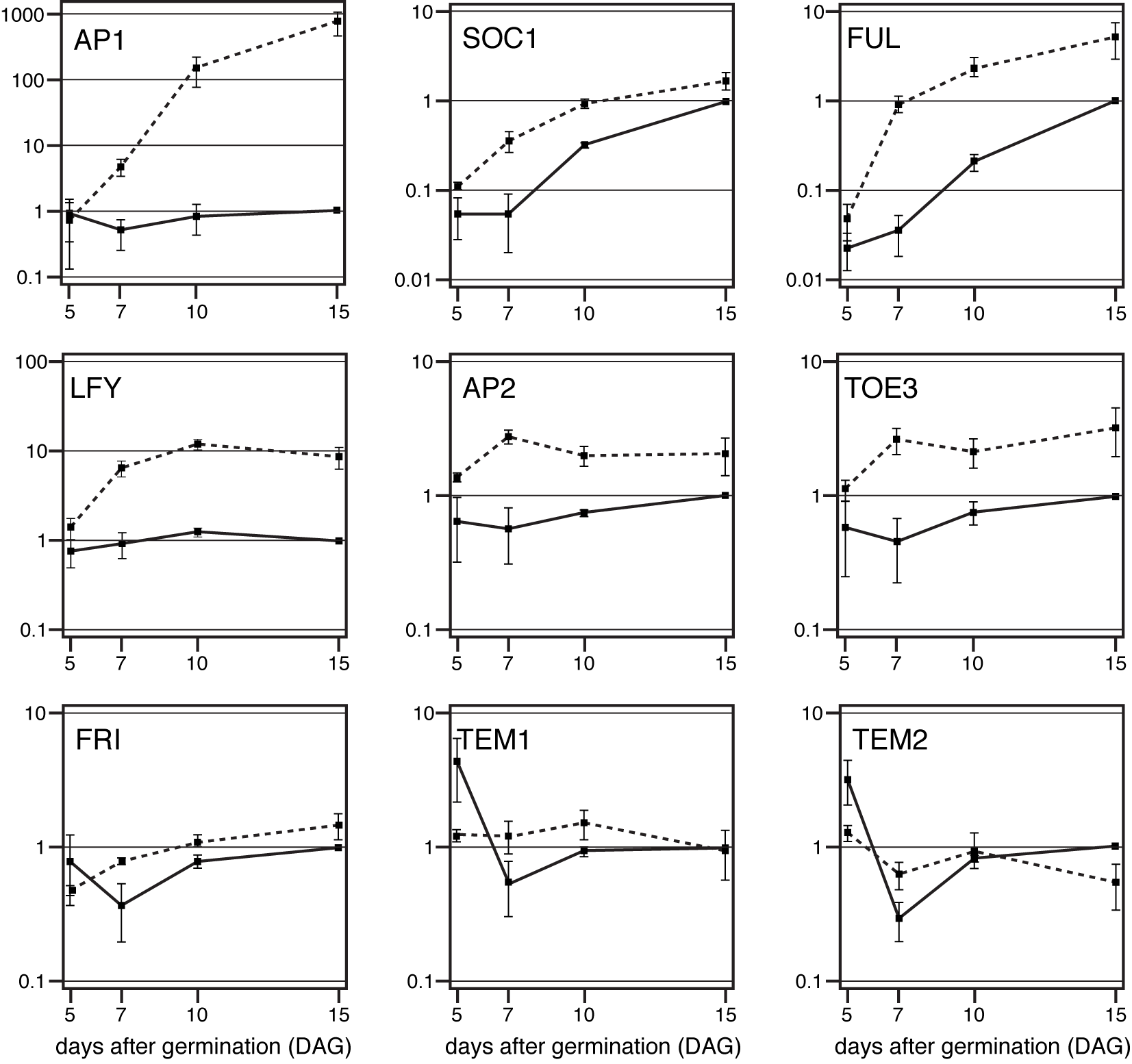

Supplement: Figure S6 — Expression of flowering time genes in the toe1 toe2 smz snz quadruple mutant. (0.39 MB TIF) [file pbio.1000148.s011.tif]
