# Supplementary material for: Repression of Flowering by the miR172 Target SMZ
Source: PLoS Biol. 2009 Jul 7;7(7):e1000148. doi: 10.1371/journal.pbio.1000148 (PMC2701598; doi:10.1371/journal.pbio.1000148)
Supplement: Table S1 — Mutant lines used throughout this work. (0.05 MB DOC) [file pbio.1000148.s012.doc]

| **Gene** | **AGI** | **Allele** | **Type** | **Source** | **Reference** |
| --- | --- | --- | --- | --- | --- |
| *SMZ* | At3g54990 | *smz-1* | insertion, intron | SM_3.22540 | this work |
|  |  | *smz-2* | insertion, intron | Salk_110654 | this work |
|  |  | *smz-D* | activation tagging |  | Schmid et al., 2003 |
| *SNZ* | At2g39250 | *snz-1* | insertion, 5’ UTR | Salk_030031 | this work |
| *TOE1* | At2g28550 | *toe1-2* | insertion, exon | Salk_069677 | Aukerman and Sakai, 2003 |
| *TOE2* | At5g60120 | *toe2-1* | insertion, exon | Salk_065370 | Aukerman and Sakai, 2003 |
| *FLC* | At5g10140 | *flc-3* | deletion |  | Michaels and Amasino, 1999 |
| *FLM* | At1g77080 | *flm-3* | insertion, exon | Salk_141971 | Sung et al., 2006 |
| *SVP* | At2g22540 | *svp-31* | insertion, exon | Salk_026551 | Lee *et al*. 2007 |
