# Supplementary material for: Repression of Flowering by the miR172 Target SMZ
Source: PLoS Biol. 2009 Jul 7;7(7):e1000148. doi: 10.1371/journal.pbio.1000148 (PMC2701598; doi:10.1371/journal.pbio.1000148)
Supplement: Table S2 — Oligonucleotides used in this work. (0.13 MB DOC) [file pbio.1000148.s013.doc]

| **ID** | **Gene** | **Sequence** | **Purpose** |
| --- | --- | --- | --- |
| *Oligonucleotides for genotyping* | | | |
| G-00989 | *SMZ* | CTG CGT AAG CAG TAT CAA ACC CGC CTG | *smz-1* |
| G-00960 |  | TTT GAT TTG TAG ATC TTC TCT GAC AAC | *smz-1* |
| G-00961 |  | AAT CAT CCA CGA CGA AAT TGA TGT CTG | *smz-2* |
| G-00960 |  | TTT GAT TTG TAG ATC TTC TCT GAC AAC | *smz-2* |
| G-00407 | *SNZ* | ATC CAA CCA CTC ATT TCC GGG | *snz-1* |
| G-00408 |  | AGG TCC CCA ACA CGT TCC ATT | *snz-1* |
| G-09565 | *TOE1* | GAA GAG TTT GTG CAT ATA CTG CG | *toe1-2* |
| G-09566 |  | GAA GGG AAG TGA AAG AGC CTC | *toe1-2* |
| G-09567 | *TOE2* | TCC CAG CAG AAA TCA GTT CAC | *toe2-1* |
| G-09568 |  | AGT TGT GCT CTA CAC GAA CGG | *toe2-1* |
| G-00868 | *FLC* | AAA ATA TCT GGC CCG ACG AAG | *flc-3* |
| G-00869 |  | CGA CGA GAA GAG CGA CGG ATG | *flc-3* |
| G-12063 | *FLM* | CGG AGA AAC CTC AAT GTT TTG | *flm-3* |
| G-12064 |  | GGT TTT GTG GAG TAA TTG GTT G | *flm-3* |
| G-01291 | *SVP* | GGA GCT ACA GAA CTC GAA CAG | *svp-31* |
| G-01292 |  | CTC ATT CTT GAA TCT TGA TCC | *svp-31* |
| G-17720 | *pROK2* | ATT TTG CCG ATT TCG GAA C | Salk T-DNA lines |
| G-03124 | *dSpm* | GGT GCA GCA AAA CCC ACA CTT TTA CTT C | SM T-DNA lines |
| *Oligonucleotides for qRT-PCR* | | | |
| N-0078 | *TUB* | GAG CCT TAC AAC GCT ACT CTG TCT GTC |  |
| N-0079 |  | ACA CCA GAC ATA GTA GCA GAA ATC AAG |  |
| G-00626 | *FT* | TCC CTG CTA CAA CTG GAA CAA CCT TTG |  |
| G-00627 |  | CGC AGC CAC TCT CCC TCT GAC AAT TGT |  |
| G-00628 | *SOC1* | ATA GGA ACA TGC TCA ATC GAG GAG CTG |  |
| G-00629 |  | TTT CTT GAA GAA CAA GGT AAC CCA ATG |  |
| G-00630 | *LFY* | AGT TCC TTC TTC AGG TCC AGA CAA TTG |  |
| G-00631 |  | CTT CTT CGT CTA GGC AGT GGA GAG CGT |  |
| G-00634 | *AP1* | AGG GAA AAA ATT CTT AGG GCT CAA CAG |  |
| G-00635 |  | GCG GCG AAG CAG CCA AGG TTG CAG TTG |  |
| G-00654 | *FUL* | TTG CAA GAT CAC AAC AAT TCG CTT CTC |  |
| G-00655 |  | GAG AGT TTG GTT CCG TCA ACG ACG ATG |  |
| G-00656 | *AP2* | GCA TAA AAG TCA AGA TAT GCG GCT CAG |  |
| G-00657 |  | GCA TAA AAG TCA AGA TAT GCG GCT CAG |  |
| G-00658 | *SMZ* | AGG GAG AAG GAG CCA TGA AGT TTG GTG |  |
| G-00659 |  | GTC TTC AGA GGT TTC ATG GTT GCC ATG |  |
| G-05431 | *FRI* | ACT GAA GGA GGA TTA GCT GCG GCT GAG |  |
| G-05432 |  | CAC GCT TGA TAC TTG ATT CAA CTA TAC |  |
| G-08107 | *TOE3* | ACG AGG AAC GGT CAT AAT CTT G |  |
| G-08113 |  | ATT CCC ACC ACT CGA TTT CCT |  |
| G-22650 | *TEM1* | ACC AGA CCG GCA ATT GTA TAT CCA C |  |
| G-22651 |  | ATC TCT TCT TGC CAA CAC ACT CTA CTG |  |
| G-22652 | *TEM2* | GAC TAG AGC GGC AGT TAT ATA TTG AT |  |
| G-22653 |  | CTT TCC ACC GCA AAC GGC CA |  |
| *Oligonucleotides for amplification of ORFs* | | | |
| G-3323 | *SMZ* | ATG TTG GAT CTT AAC CTA AAG ATC |  |
| G-5638 |  | CTA TGG ATC AAA ACA ATT GGA C |  |
| G-2050 | *rSMZ* | CAG TTG CAG CCA GCT CCG GAT TCC CTT TTA TCA GCA TG | SDM of *SMZ* cDNA^1^ |
| G-2051 |  | AAG GGA ATC CGG AGC TGG CTG CAA CTG TCT TCA GAG GT | SDM of *SMZ* cDNA^1^ |
| G-16615 | *SMZ* | GGA TCC GGA AGT GGT TCG GGC AGC GGA AGT TTG GAT CTT AAC CTA AAG A | *GS_10_:SMZ^2^* |
| G-18665 |  | GGG AGT GGT TCA GGA AGT GGG TCT GGC TCA GGA TCC GGA AGT GGT TCG GGC AGC | *GS_10_:SMZ^2^* |
| G-16616 |  | CTA TGG ATC AAA ACA ATT G | *GS_10_:SMZ^2^* |
| G-08237 | GUS | CAC CGG TAT GGT CCG TCC TGT AGA AAC C |  |
| G-08238 |  | GAC CGG TTC ATT GTT TGC CTC CCT GCT GCG |  |
| *Oligonucleotides for ChIP Confirmation* | | | |
| G-23055 | FT (pos) | CCA AGT GTA ATG ATG TCC ACG | FT ChIP |
| G-23056 |  | CCT ACT ACA AAA GTA GTC GAG GCG | FT ChIP |
| G-23057 | FT (neg) | GGT TCT TTC ACT TGA ACT CCC | ChIP neg. control |
| G-23058 |  | CCA AGG TCT CTG CAT GCC CAA G | ChIP neg. control |
| G-23145 | TOE3 | gtg tgt cgg cac aat cat ctc | TOE3 ChIP |
| G-23146 |  | CCG TCC GAT GTT TCC AAG CTG | TOE3 ChIP |
| G-23544 | SNZ | gtg cat atc tca acc gtc ca | SNZ ChIP |
| G-23545 |  | cca tat acg gaa agg cca ga | SNZ ChIP |
| G-23546 | SMZ | Caa agg aca acc ctt ttc ca | SMZ ChIP |
| G-23547 |  | ata ttg gga tgc gaa agc ac | SMZ ChIP |
| G-23548 | AP2 | Gtc ggt gtc gaa gga tgt tt | AP2 ChIP |
| G-23549 |  | ata cgt ttt gcg gtc caa ag | AP2 ChIP |
| G-23550 | TEM1 | ggg ggt tca cat gag att tg | TEM1 ChIP |
| G-23551 |  | tga tca aga cct ccc caa ct | TEM1 ChIP |
| G-23552 | FRI | tgc cgt gaa tat acc acc aa | FRI ChIP |
| G-23553 |  | aaa acg tag ccg ttg att cg | FRI ChIP |
| G-23556 | SOC1 | Aca tca acg tcg tcc gta ca | SOC1 ChIP |
| G-23557 |  | Tgt ctc ggg att ttt cga tt | SOC1 ChIP |
| G-2073 | AP1 | GCA AAG CGA AGG TGA CAC | AP1 ChIP |
| G-2074 |  | CGT ACT AAT GTC GGG TCC | AP1 ChIP |

1) SDM = site directed mutagenesis 2) GS_10_ = adding 10 Gly-Ser pairs to SMZ ORF
